# Role of the salt glands of *Armeria maritima* (halophyte) in removal of lead from tissues

**DOI:** 10.1007/s11356-024-33624-z

**Published:** 2024-05-24

**Authors:** Małgorzata Wierzbicka, Martyna Begiedza, Karolina Bodzon, Olga Bemowska-Kałabun, Krzysztof Brzost, Monika Wróbel, Damian Trzybiński, Krzysztof Woźniak

**Affiliations:** 1 Department of Ecotoxicology, Institute of Environmental Biology, Faculty of Biology, University of Warsaw, Miecznikowa 1, 02–096 Warsaw, Poland; 2 Faculty of Chemistry, Biological and Chemical Research Centre, University of Warsaw, Żwirki i Wigury 101, 02–089 Warsaw, Poland

**Keywords:** *Armeria maritima* (Mill.) Wild, Halophyte, Lead, Plant crystals, Crystal structure, Phytoremediation

## Abstract

*Armeria maritima* is a halophyte exhibiting a strong tolerance to heavy metals. It grows on zinc–lead waste heaps. This study aimed to determine the role of salt glands in the removal of lead (Pb) from plants and to trace the path of lead from the shoots to the salt glands on the surface of leaves. Mechanisms allowing high tolerance to lead in *A. maritima* were also evaluated. These examinations were conducted on a lead-tolerant population and a lead-sensitive plant population. The plants were treated with Pb(NO_3_)_2_ and the path of lead was traced from the roots to the leaves. The lead-tolerant population transported twice as much lead as the sensitive population. The action of the salt glands resulted in 40% of the leaf lead content in the lead-tolerant population being expelled onto the surface of the leaves. These features indicate the high phytoremediation capabilities of these halophyte plants. The excretion of multi-ionic solutes by the salt glands results in the appearance of tiny crystals on the surface of the leaves. In this publication, for the first time, an attempt was made to determine what chemical compounds build up these crystals and to determine their crystal structure. Solving this problem was possible through the usage of single-crystal X-ray structural analysis.

**Supplementary Information:**

The online version contains supplementary material available at 10.1007/s11356-024-33624-z.

## Introduction

Lead is a strongly toxic heavy metal which, having penetrated a plant, may cause inhibition of its growth, cell divisions and chlorophyll production (Wierzbicka 1995a, 1995b; Ghorbani et al. 2024, 2018). The toxic action of lead is caused by cell membrane damage, which leads to disordered membrane permeability (Sharma and Dubey 2005; Grzesiuk et al. 2011). After Pb^2+^ ions make their way into a cell, cytoplasm proteins undergo denaturation, which results in cell death (Wierzbicka 1995a, 1995b; Przedpełska–Wąsowicz and Wierzbicka 2015). Soil lead (Pb) concentrations may exceed 1000 mg/kg in heavily industrialised areas although the geochemical background concentration is usually 18 mg/kg (Lis and Pasieczna 1995; Verma and Dubey 2003; Cegiełkowska et al. 2015; Wierzbicka 2015).

Despite the high toxicity of lead, there are certain plant species (metallophytes (Whiting et al. 2004)) that are particularly lead-tolerant which can grow in post-industrial areas such as zinc–lead waste heaps. *Armeria maritima* (Mill.) Wild. of the Plumbaginaceae family belongs to this group (Abratowska et al. 2012; Dahmani–Muller et al. 2000). These are perennial herbaceous plants which form sods (Rutkowski 1998). The microevolutionary adaptation of this plant species has been reported by Wierzbicka et al. (2023). The characteristic feature of *A. maritima* is that it has salt glands on the surface of its leaves which serve to excrete salt when the soil has excess salt. This is an adaptation typical of halophytes or plants growing in salinized soils, such as at the seaside. Salt glands are built from a group of cells specialised for removing sodium chloride (NaCl). These secretory structures are present in numerous species (*N* = 45) belonging to the Plumbaginaceae family.

This paper aimed to trace the Pb^2+^ pathway in *A. maritima* plants to investigate the mechanisms behind their exceptionally high tolerance to lead and the role of salt glands in removing lead from leaf tissues. However, research on *A. maritima* provides also a great opportunity to better understand the phenomenon of crystal formation by plants, as this plant is able to produce such material. The appearance of tiny crystals on the surface of its leaves as a result of the excretion of solutes by salt glands was noted almost 30 years ago, but the description of this phenomenon was limited to the statement that besides P, S, Cl, K and Ca elements the crystals contain large amounts of Cu and, to a smaller extent, Zn, Ni, Fe and Mn (Neumann et al. 1995). It is worth emphasising that this information was obtained using energy dispersive X-ray spectroscopy, an analytical tool limited to surface analysis due to its shallow penetration depth (Goldstein 2003). Until this moment, the documented state of knowledge has not significantly changed. Studies on the secretion of crystals by plants have been generally focused on their elemental composition and description of their morphology (He et al. 2012; Cuéllar-Cruz et al. 2020). Of course, there is no doubt that this type of research is valuable as the production and function of crystals in the plant kingdom are still far from being fully understood (Cuéllar-Cruz et al. 2020). However, in such studies, information on short- and long-range structural order of ions/molecules within a given sample is still inaccessible. It is worth noting that inorganic systems can also exist in different crystalline phases for the same compound (i.e. polymorphism). In such cases, two or more crystals have the same quantitative and qualitative composition, but ions and molecules within them exhibit different spatial arrangements (Brog et al. 2013). However, even simple inorganic salts may be hydrated, and the number of water molecules in particular crystalline forms can differ significantly (Schmidt et al. 2014). These issues can be elucidated by diffraction methods, in particular, X-ray diffraction (Waseda et al. 2011; Shih 2013; Ladd and Palmer 2013). In this paper, we decided to go further and determine the exact chemical compounds forming these crystals and establish their crystal structure.

## Materials and methods

### *A. maritima* plant cultivation

The study was conducted on *A. maritima* plants (Mill.) Wild. The seeds were taken from two different populations of *A. maritima*:

- A lead-sensitive (natural) population, which was not affected by industrial contamination. Seed collection from Kamieńczyk in the Wyszków area (Mazowieckie Province), Poland.
- Lead-tolerant population, that is plants from the zinc–lead waste heap containing heavy metals (lead (2500 mg/kg), zinc, cadmium and thallium). The waste heap was located near a non-ferrous foundry in Bolesławiec in the Olkusz area (Małopolskie Province), Poland.

The *A. maritima* plants were cultivated in two distinct manners.

*A. Cultivation of the plants in soil to obtain leaves to be treated with lead for 24 h (method 1)*

The seeds of both *A. maritima* plant populations were cultivated in a greenhouse at 25 °C/20 °C with a photoperiod of 16 h/8 h (day/night) for 6 months. The study was conducted on the leaves cut at the base of 6-month-old plants. The leaves were sampled from the middle whorl. Subsequently, the leaves were placed for 24 h in 5 mM, 10 mM or 20 mM lead nitrite solutions (Pb(NO_3_)_2_) A control group of leaves was placed in distilled water. Each experimental combination involved 16 cultivated plants, totalling (4 combinations × 16 plants) 64 plants.

*B. Cultivating plants in perlite and treating them with lead for 6 months (method 2)*

The *A. maritima* seeds from both populations were sprouted on a moist filter paper in Petri dishes at 15 °C. After sprouting, the seedlings were transferred to a greenhouse and placed in perlite-filled containers. Knop’s medium was used at 1/2 dilution, containing Ca(NO_3_)_2_, KNO_3_, KCl, hydrated MgSO_4_ and EDTA–Fe (Knop 1865). Three micromolar lead was added to the medium as Pb(NO_3_)_2_. The lead-containing medium was replaced every 2 weeks. Cultivation took 6 months and covered the full lifecycle of the plants. Control plants were cultivated in perlite in lead-free medium, replaced every 2 weeks. Each experimental combination involved 16 cultivated plants, totalling (8 combinations × 16 plants) 128 plants.

### Visualisation of lead in leaf tissues

Plants cultivated by method A were studied to visualise lead in the leaf tissues. The histochemical method was applied with dithizone (diphenylthiocarbazone) staining (Seregin and Ivanov 1997). When this organic compound was combined with Pb^2+^ ions, the dark green colour of the solution changed to red or dark brown (Lipiec and Szmal 1980). Cross sections were made through the central part of the leaf and the sections were placed in a dithizone solution for 24 h (in the dark). The location of lead deposits in leaf tissues were observed with a Nikon EFD 3 optical microscope. One thousand samples were examined in total.

### Ultrastructural visualisation of lead

A transmission electron microscope (TEM) with energy dispersive X-ray (EDX) microanalysis was used to examine the places where lead was located at subcellular level. The leaves of the plants cultivated by method A were selected for microscope study. The leaves were incubated in the Pb(NO_3_)_2_ solution at the 20 mM concentration for 24 h. Two spots on every leaf were analysed: the middle section of the leaf and the lower epidermis in the middle section of the leaf. All leaf fragments were rinsed in an ultrasonic washer (made by POLSONIC) in a cycle of 2 × 10 s to remove lead from the plant surface before the leaf fragments were fixed (Antosiewicz and Wierzbicka 1999). X-ray microanalysis was performed on the sections that were not stained with Reynolds’ reagent (1963) as the reagent contains lead. Observations of ultrathin sections were conducted with a JEOL JEM 1400 high-resolution transmission electron microscope with a digital camera. One hundred samples were examined in total.

### Lead concentrations in plants

The plants cultivated by method B were examined. After treating them with 3 µM Pb(NO_3_)_2_ for 6 months, the Pb^2+^ concentration was measured in the roots and shoots. Roots and shoots were weighed separately. The shoots of each studied plant were divided into two sets. One set of the shoots was washed in distilled water in a POLSONIC ultrasonic washer to remove lead and other elements from the plant surface, and the other part was not washed at all. Subsequently, the samples were dried at 65 °C until solid. The dried leaves were milled with an agate ball mixer mill (Retsch MM200) and subjected to mineralisation in 69% nitric acid (HNO_3_). The lead content in the samples was determined with atomic absorption spectrometry (AAS) using a CONTRAA 300 spectrometer. Virginia Tobacco Leaves CTA-2 purchased from Institute of Nuclear Chemistry in Poland were used as a reference. Two hundred fifty-six samples were examined in total.

### Examination of crystals on the leaves

*A. maritima* leaves were examined for the appearance of crystals on their surfaces during 6 months of cultivation in mineral medium with lead (method B). Four mature leaves were cut off each plant. Observations were conducted on dry leaves in a scanning electron microscope (SEM) with X-ray microanalysis (EDX) – using a Phenom ProX instrument. Four hundred twenty-eight samples were examined in total.

Clusters of crystals (rarely distinct single-crystals) were extracted from the spots on the leaves as described above and investigated using electron microscopy and then transferred to drops of paratone-N oil in Petri dishes. The extraction process was carried out using an acupuncture needle dipped in the oil. Individual crystals were separated from the clusters using a scalpel or a thin steel needle. Selected crystals were then mounted in paratone-N oil using MiTeGen micro-loops and subjected to the preliminary X-ray diffraction experiments using the Agilent Technologies SuperNova Dual Source diffractometer at 100(2) K using Cu*K*α (*λ* = 1.54184 Å) radiation. Diffractometric screening initially assessed the quality of investigated samples and allowed for determining their lattice parameters (in cases where it was possible). Those parameters were obtained by least-squares fitting to the optimised setting angles of the reflections collected using CrysAlis Pro software (Oxford Diffraction 2008). After establishing the complete set of unit cell parameters, full X-ray diffraction measurements were carried out for selected representatives. Data were reduced using CrysAlis RED and used to establish the crystal structure of chemical compounds forming the crystals (Oxford Diffraction 2008). In all cases, multi-scan empirical absorption correction using spherical harmonics, implemented in SCALE3 ABSPACK scaling algorithm, was applied (Oxford Diffraction 2008). The structural determination procedure was carried out using the SHELX package (Sheldrick 2008). The structures were solved with intrinsic phasing, and then successive least-squares refinements were carried out based on full-matrix least-squares on *F*^2^ using the SHELXL (version 2014/7) program (Sheldrick 2008). The hydroxyl H-atoms of water molecules in the case of gypsum, picromerite and calcium sulphate subhydrate were located on the Fourier difference electron density map and refined with the O–H bond length restrained to 0.85 Å, with *U*_iso_(H) = 1.5*U*_eq_(O). The H···H distance within the water molecule was additionally restrained to 1.39 Å. The hydroxyl H-atoms in syngenite were positioned geometrically with O–H bond length equal to 0.82 Å and constrained with *U*_iso_(H) = 1.5*U*_eq_(O). Chemical occupancy for the O1W atom of the water molecule in the crystal of the investigated calcium sulphate subhydrate was refined freely (final site occupancy factor value for O1W atom was 0.69(3)). The above O-atom was located on the twofold axis which resulted in disorder of water H-atoms over two respective positions with the occupancies of 0.347(14) for each of those atoms. Figures were prepared using Olex2 and Mercury (Dolomanov et al. 2009; Macrae et al. 2020).

## Results

### Leaves treated with lead (method 1)

Cut leaves were treated with lead in order to trace the pathway of lead and its location in tissues and cells (method A). Note that after that time of lead treatment, no abnormalities in leaf morphology were found compared to the controls. Determination of the applied lead dose was preceded by numerous preliminary examinations to select the appropriate dose (time and concentration). It was also vital that the concentration not be lethal. Ultimately, a relatively short incubation time (24 h) and lead concentrations of 5–20 mM were chosen. The selected incubation time was so short that lead managed to penetrate only into some tissues. This allowed assessment of the translocation rate of lead and its pathway in the leaves. Under such circumstances, the lead concentration in leaves was 50 mg/kg of dry mass – after 5 mM lead treatment for 24 h. This was an amount comparable to the results obtained after 6-month treatment of plants with lead (0.003 mM) (method 2), where the final lead concentration in leaves was 50–100 mg/kg of dry mass. Based on this, it was determined that the results obtained in both experiments would be comparable.

### Visualisation of lead in leaf tissues

Microscopic observations employing dithizone staining allowed the location of lead deposits in leaf tissues. As no differences in lead distribution were found between the two populations, it was concluded that the presented results concern the leaves of one of the populations—the lead-tolerant one.

**Fig. 1:**
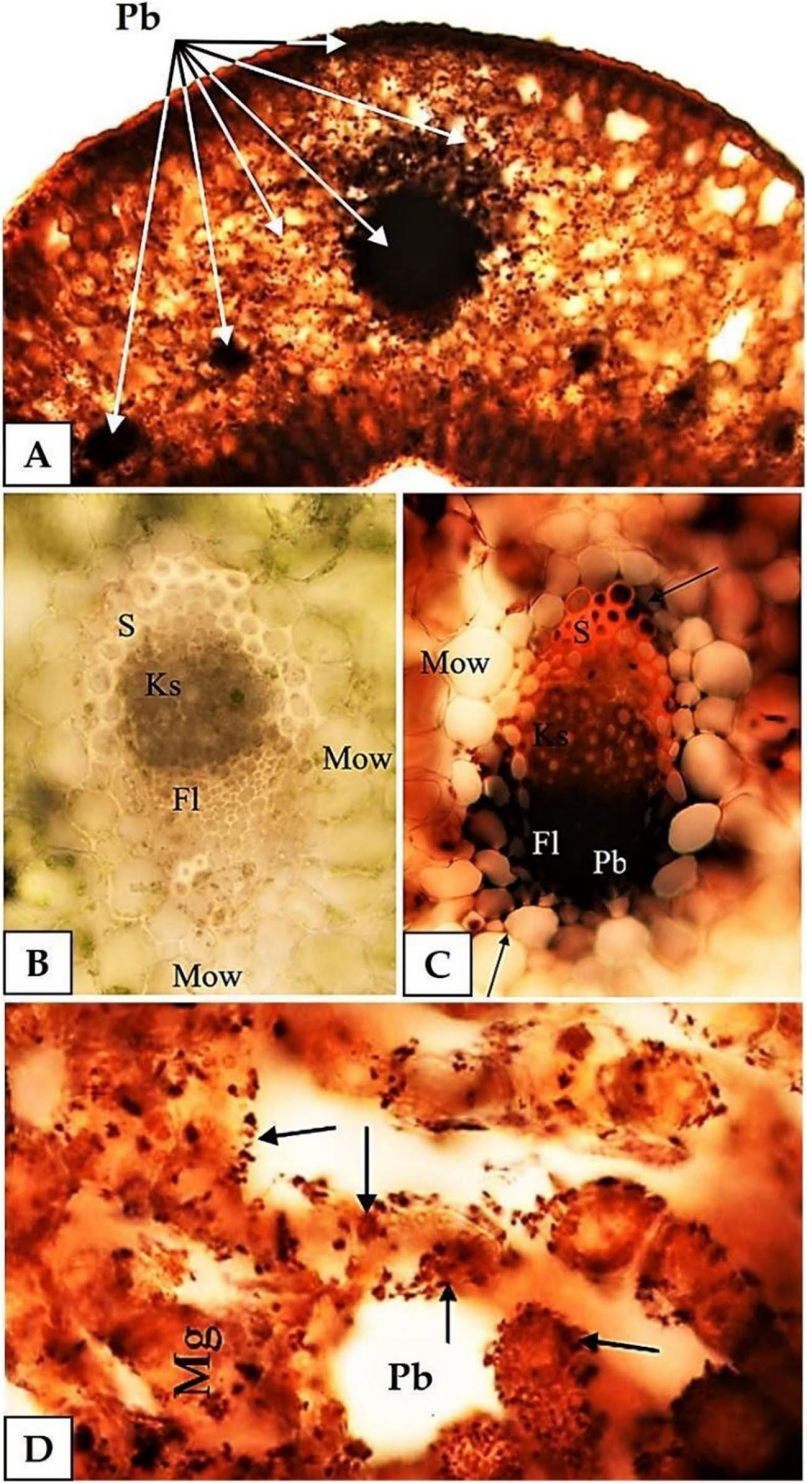
Visualisation of lead in *A. maritima* leaves—cross sections, dithizone staining, lead concentration at 10 mM; optical microscope. **A** Lead in leaf tissues (Pb), in epidermis, vascular bundles of the mesophyll, arrows, 100 × magnification. **B** Controls—vascular bundle, no lead, sclerenchyma (S), xylem (Ks), phloem (Fl), mesophyll (Mow), 400 × magnification. **C** Vascular bundle with lead (Pb), the highest amounts of lead visible in phloem (Fl), 400 × magnification. **D** Lead in intercellular spaces and on cell surfaces (Pb), 400 × magnification

When observing the cross sections of the leaves (Fig. 1), it was found that the highest amounts of lead were present in vascular bundles (Fig. 1A), in the phloem in particular (Fig. 1B, C). The intensity with which the vascular bundles were stained dark brown increased as the Pb^2+^ concentration in incubation solutions went up. This result indicates increased Pb^2+^ concentrations in plant tissues. Considerable amounts of lead were also found in intercellular spaces of the mesophyll (Fig. 1D), but only in the vicinity of the vascular bundle (up to 5 cell layers). The intercellular spaces of the leaf mesophyll are exceptionally large in *A. maritima* and are able to store considerable amounts of lead (Fig. 1D).

**Fig. 2:**
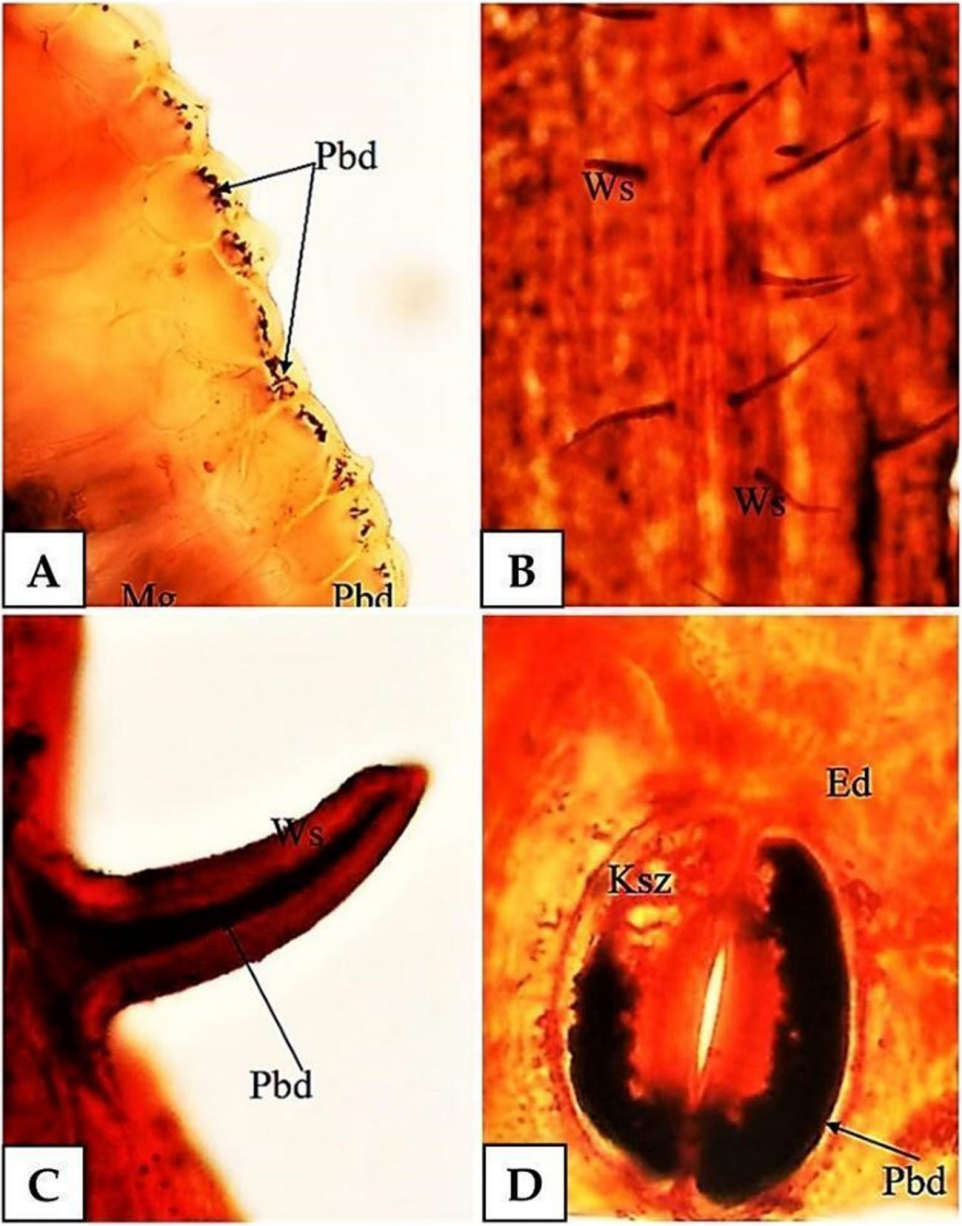
Lead visualisation in the epidermis of *A. maritima*, dithizone staining, lead concentration at 10 mM; dithizone staining, optical microscope. **A** Lead in epidermal cells (Pbd), 200 × magnification. **B**, **C** Lead in hairs (Ws) (**B** 200 × magnification; **C** 400 × magnification). **D** Lead in parastomal cells (Ksz) of the stoma (Pbd) (Ed) epidermis, 400 × magnification

During the visualisation of lead in the leaf epidermis, it was observed that considerable lead deposits occur both in the epidermis (Fig. 2A) and in all epidermal products: epidermal hairs (Fig. 2B, C) and stomas (Fig. 2D).

**Fig. 3:**
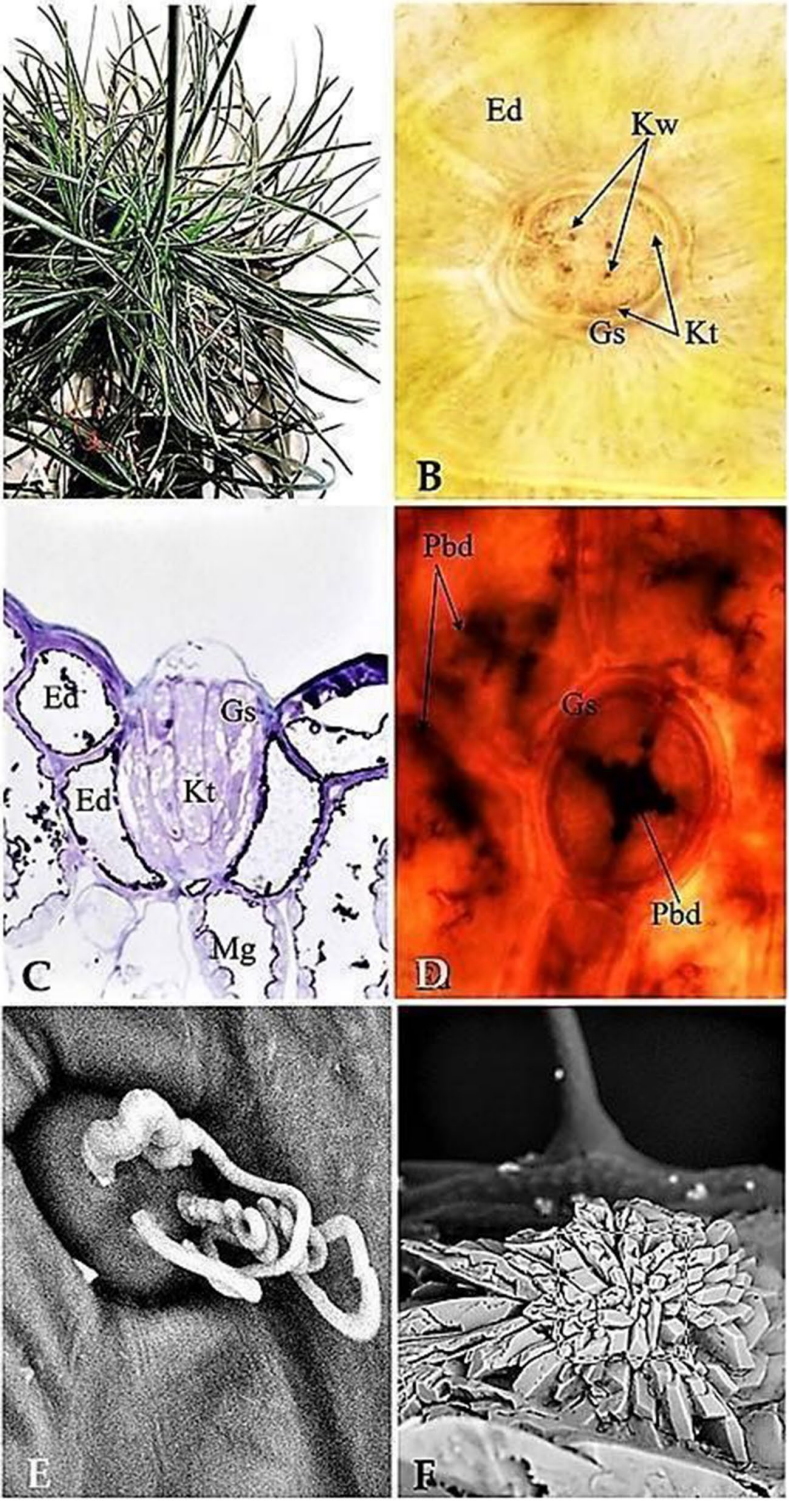
Salt glands in *A. maritima* plants. **A** General exterior of the *A. maritima* plant—leaf rosette. Plant age: 6 months. **B**, **C** Controls—salt gland (Kw, Kt) between epidermal cells (Ed), salt-gland secretory cells (Kt), salt secretion sites (Kw) (**B** leaf surface; optical microscope; 400 × magnification; **C** cross section through a salt gland, electron microscope, 1200 × magnification). **D** Lead (Pbd) in a salt gland (Gs); optical microscope; lead at 10 mM for 24 h, dithizone, 400 × magnification. **E** Salt secretion of a salt gland; scanning electron microscope, lead at 0.003 mM for 6 months, scanning electron microscope, 10,000 × magnification. **F** Conglomerate of salt crystals on the surface of a leaf above a salt gland; lead at 0.003 mM for 6 months, scanning electron microscope, 6000 × magnification

*A. maritima* can be characterised by the presence of salt glands (Fig. 3). They are distributed among epidermal cells (Fig. 3B). The structure of the gland is shown in Fig. 3C. After treating the leaves with Pb^2+^, lead was detected in the salt glands (Fig. 3D). The main role of the salt glands is to secrete excess salt (Fig. 3E), which resulted in crystals forming on the surface of leaves (Fig. 3F).

### Ultrastructural visualisation of lead in leaf cells

**Fig. 4:**
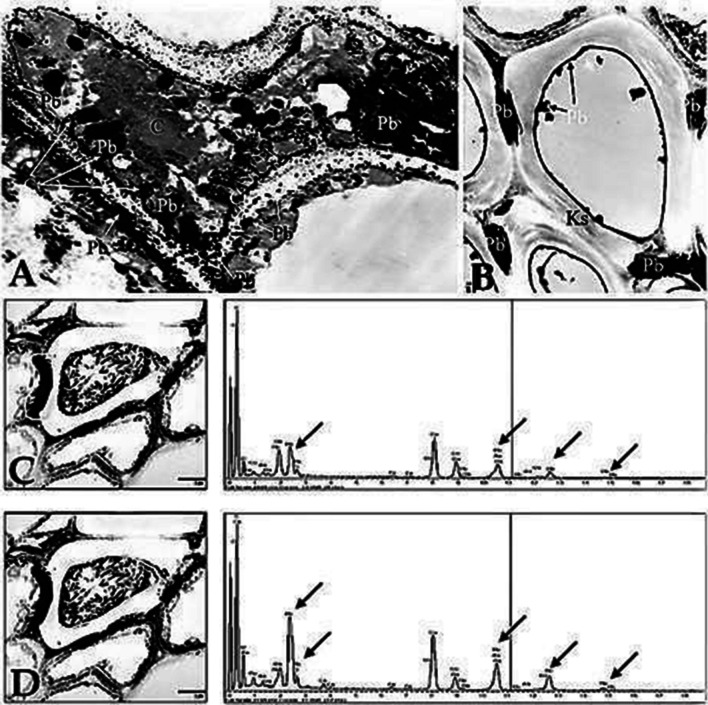
Lead in a vascular bundle, lead concentration at 20 mM, electron microscope. **A** Phloem with electron-dense deposits (Pb) in transfer walls; 8000 × magnification. **B** Xylem (Ks) with electron-dense deposits in the lumen of a vessel (arrows), deposits in a cell cavity between vessels (Pb); 8000 × magnification. **C**, **D** Electron-dense deposits in xylem cells. X-ray microanalysis: **C** in the cell lumen; **D** in a cell wall. Lead (Pb) was marked with arrows on X-ray microanalysis spectra, 8000 × magnification

**Fig. 5:**
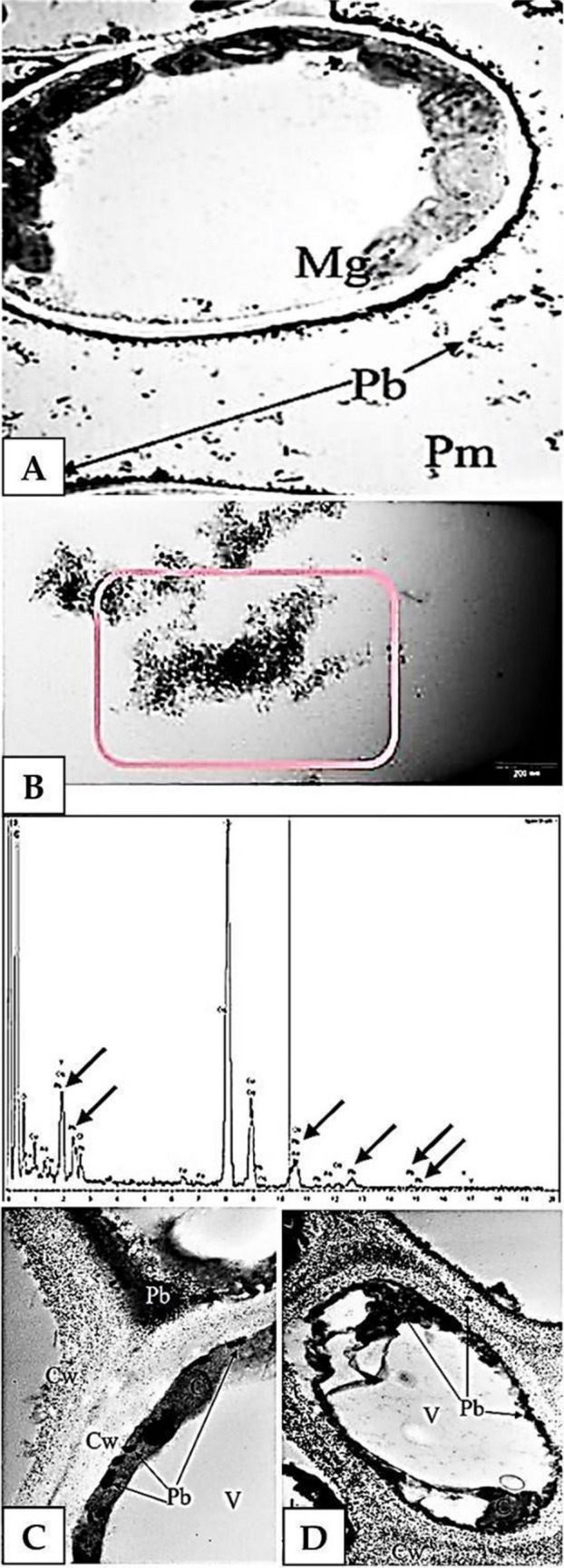
Lead (Pb) in mesophyll cells, lead at 20 mM, electron microscope. **A** Intercellular spaces (Pm) between mesophyll cells (Mg), electron-dense deposits (Pb); 8000 × magnification. **B** X-ray microanalysis of a deposit marked in red in the intercellular space, lead marked with arrows on the microanalysis spectrum. **C** Fragment of three mesophyll cells, electron-dense deposits (Pb) in a cell wall (CW), cytoplasm (V), vacuole, 8000 × magnification. **D** Maturing sclerenchyma cell; electron-dense deposits (Pb) in a cell wall (PW), cytoplasm (C) and vacuole (V), 8000 × magnification

Observations of *A. maritima* leaves using a transmission electron microscope showed that treating the leaves with a 20 mM Pb(NO_3_)_2_ solution did not cause any damage in cell ultrastructure compared to the controls. However, after lead treatment, the presence of electron-dense deposits in cells was noted. The deposits were subjected to X-ray microanalysis. It was demonstrated that they contained lead (Figs. 4 and 5). The greatest number of deposits was found in the vascular bundle (Fig. 4). In phloem cells, deposits were observed in transfer walls and in cytoplasm (Fig. 4A). In the xylem, deposits were found both in the cell walls and the cell-connecting cavities (Fig. 4B).

In mesophyll cells, electron-dense deposits were found in the intercellular spaces of the cells (Fig. 5A, B), but only in the vicinity of the vascular bundle (up to 5 cell layers). Lead was found in those deposits (Fig. 5B). In some cells, the deposits were found along the entire circumference of the cell wall (Fig. 5C). Large structures of the deposits were observed in the cytoplasm (Fig. 5C). These deposits were also noted in sclerenchyma cell walls (Fig. 5D).

**Fig. 6:**
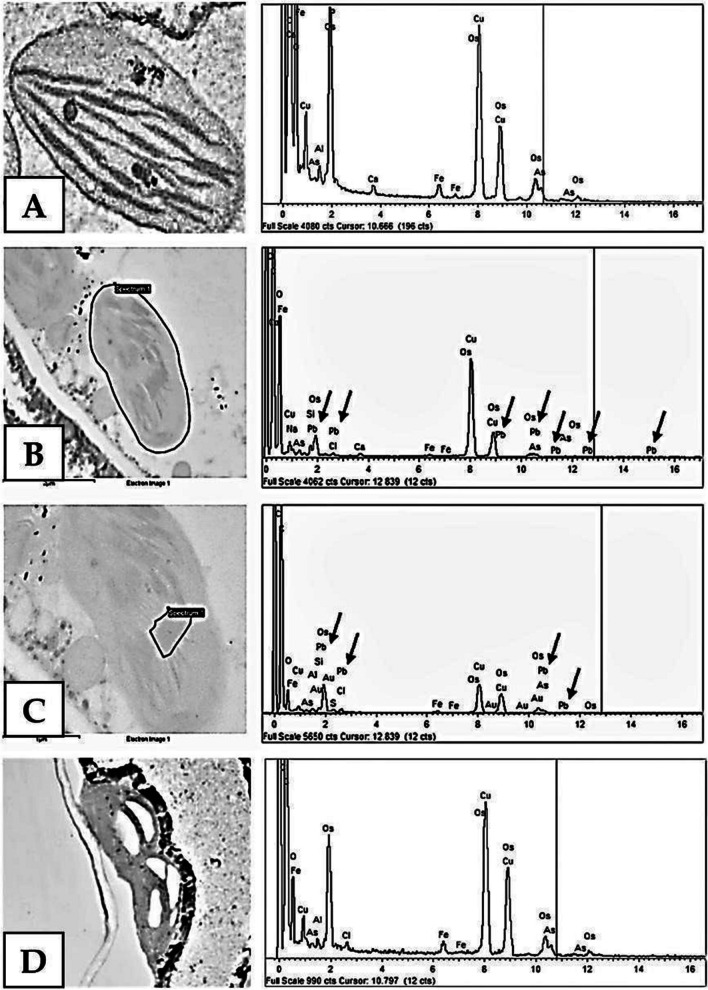
Chloroplasts in mesophyll cells. Lead at 20 mM; electron microscope, 20,000 × magnification. **A** Control—the microanalysis spectrum did not show lead presence. **B**, **C** Chloroplast in the second mesophyll cell layer, lead marked with arrows on the X-ray microanalysis spectrum: **B** microanalysis of the entire chloroplast, marked in red, **C** microanalysis of the chloroplast stroma, marked in red. **D** Chloroplast from the ninth mesophyll cell layer. X-ray microanalysis spectrum did not indicate the presence of lead

In mesophyll cell chloroplasts, lead was found present both in thylakoids and stroma (Fig. 6B, C). X-ray microanalysis was performed both in the chloroplasts of mesophyll of leaves of controls (Fig. 6A) and in the chloroplasts of leaves treated with Pb^2+^. The presence of lead was only observed in the chloroplasts of cells located close to the vascular bundle—up to 5 cell layers (Fig. 6B, C). On the other hand, lead deposits were not found in the cells located further from the vascular bundle and the chloroplasts of these cells (Fig. 6D). Given the fact that the leaves were treated with Pb^2+^ for 24 h, the calculated rate of lead transfer from the vascular bundle to individual mesophyll layers was 4.8 h per cell layer.

### Crystals on leaves

Crystals on the *A. maritima* leaves were examined on the plants cultivated for 6 months (method B) in a mineral medium with 3 µM lead. Generative shoots developed after 12 weeks of cultivation in all of the studied plants. However, the number of these shoots was lower in the plants treated with Pb^2+^: 1⎼2 shoots per plant, compared to 6⎼8 shoots per plant in the controls. Ultimately (after 6 months), the dry mass of the above-ground parts of the plants treated with Pb^2+^ was 2⎼4 times lower compared to the controls (0.2⎼0.4 g and 0.4⎼1.5 g, respectively). No statistically significant differences between the populations of plants treated with lead were found. The Pb^2+^ concentration in the leaves of those plants was 102 mg/kg of dry mass in the lead-tolerant population and 48 mg/kg of dry mass in the lead-sensitive population (Fig. 9). In the case of roots, lead accumulated at a higher concentration, namely 534 mg/kg of dry mass and 78 mg/kg of dry mass, respectively. Therefore, the root-to-shoot transport of lead was twice as high in the lead-tolerant population.

Crystals appeared on the surface of the leaves throughout the plant cultivation period. It was found that the crystals appeared on the oldest leaves on the first days of the cultivation. This phenomenon occurred at the same time in both analysed populations. However, the populations started to differ further into the cultivation period. In the case of younger leaves, the crystals appeared on lead-tolerant plants in the second week of cultivation. In the lead-sensitive population, they appeared in the third week of cultivation.

### X-ray microanalysis of crystals on the leaves

**Fig. 7:**
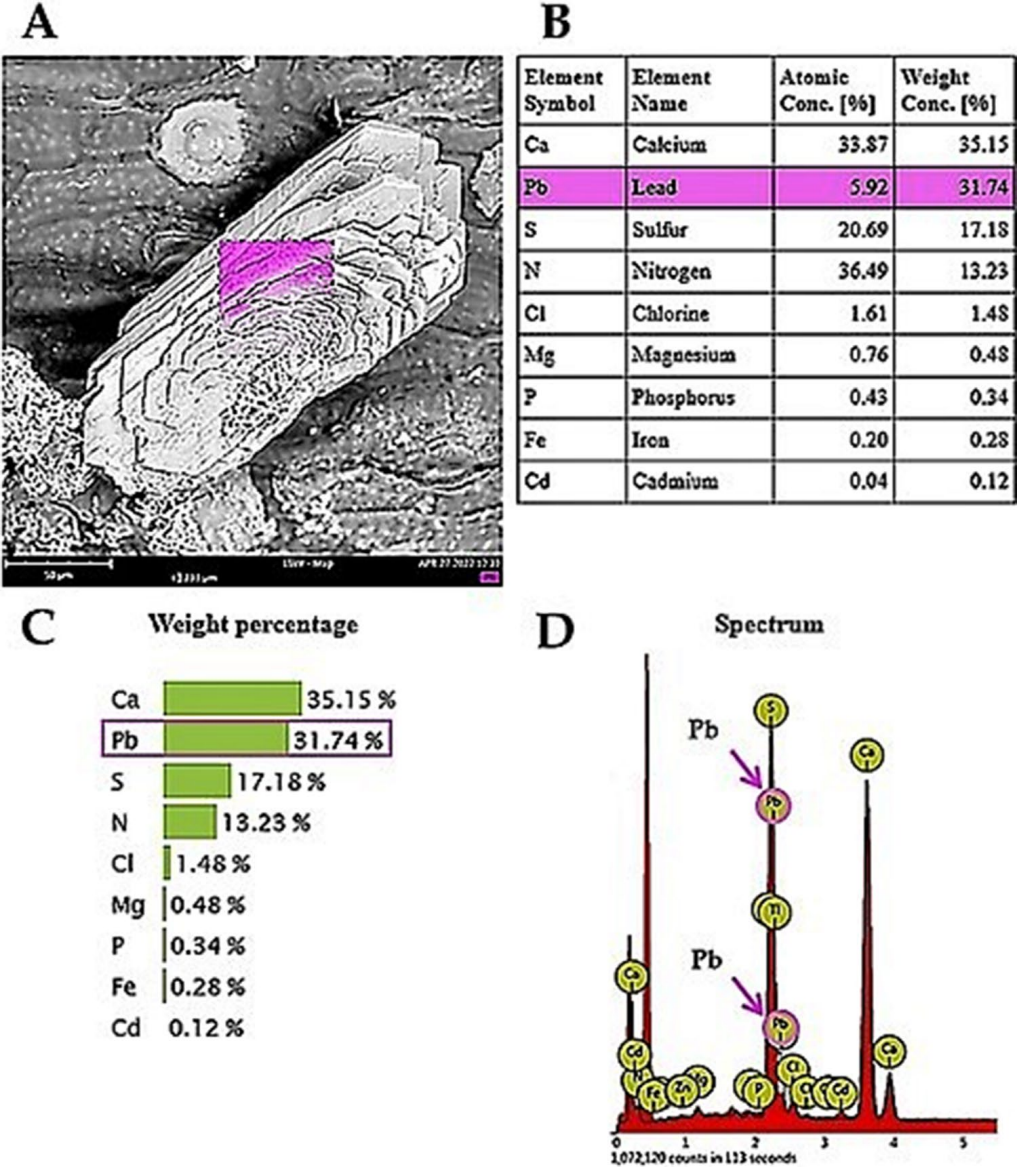
Cluster of gypsum crystals on a leaf of a plant treated with lead, concentration of 3 µM for 6 months, X-ray microanalysis, scanning electron microscope. **A** Crystal on a leaf, the coloured square marks the spot of microanalysis. The intensity of the red colour indicates lead distribution. **B**, **C** Element composition of the sample. **D** X-ray microanalysis spectrum, lead marked with arrows

**Fig. 8:**
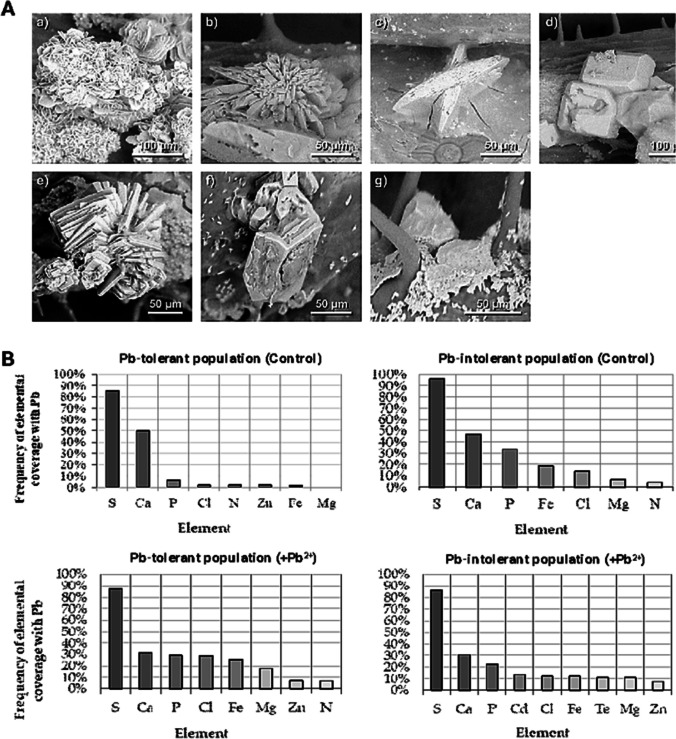
Crystals on leaves: **A** Typical morphologies of crystals from the surface of *A. maritima* leaves, where: calcium sulphate dihydrate: thin plates clustered in a “desert rose” motif (a), druse of lenticular plates (b), twinned lenticular plates; potassium nitrate: well-developed prismatic orthorhombic blocks (d); potassium calcium sulphate monohydrate: cluster of monoclinic tabular plates; (e) potassium magnesium sulphate hexahydrate: prismatic monoclinic blocks (f); calcium sulphate subhydrate CaSO_4_·0.69H_2_O: poorly developed pseudo prismatic trigonal blocks (g). **B** Element composition of crystals in the lead-sensitive population and lead-tolerant population – controls (charts on the top) and lead-treated plants (charts on the bottom), lead at 0.003 mM for 6 months. The measurements were made with X-ray microanalysis following the method illustrated in Fig. 7

The structures appearing on the leaves were examined with an electron microscope by means of X-ray microanalysis (Figs. 7, 8). The structures assuming the crystalline form were selected for the analysis (Figs. 7A, 8A). Both the percent composition of the elements (Figs. 7B, C, 8B) and the microanalysis spectra were analysed (Fig. 7D). In the vast majority of these analyses, the leaves of the lead-treated plants were observed to have lead on the surface of the examined structures (Fig. 7). The microanalyses performed on crystals found on the leaves of the lead-intolerant population showed the presence of lead in 95(± 3)% of examined crystals and in 97(± 3)% of examined crystals in the case of the lead-tolerant population. The crystals contained other elements (mostly sulphur (S) and calcium (Ca)) as well, both in the population treated with Pb^2+^ and in the controls (Fig. 8B). These elements were detected in the highest amounts in the lead-intolerant population of plants treated with Pb^2+^ (Fig. 8B). Other elements detected in the examined samples in small amounts were phosphorus (P), nitrogen (N), magnesium (Mg), chlorine (Cl) and potassium (K).

These results allow the relative comparison of the elements on the surface of the crystalline material found on *A. maritima* leaves in both populations and show that the crystals on the lead-tolerant plants treated with lead contained the highest amounts of these elements (Fig. 8B, charts on the bottom).

### Effectiveness of expulsion of lead through salt glands

**Fig. 9:**
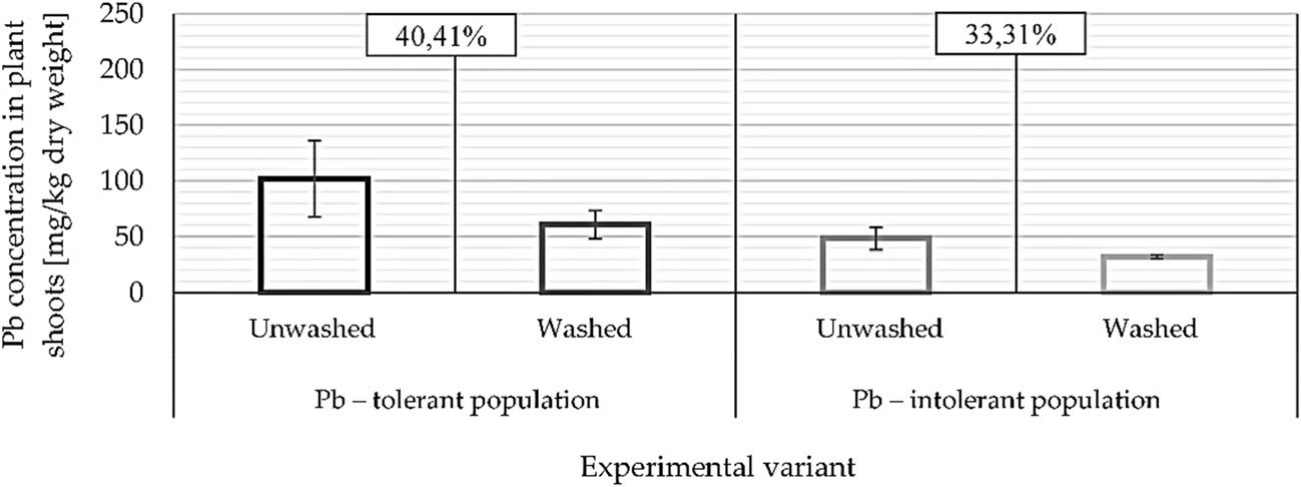
Lead concentration in leaves of lead-treated plants in the lead-tolerant population and the lead-intolerant population. The leaves were not washed or were washed in water before analysis. The amount of lead washed off the surface of the leaves was determined (40.41%: 33.31%)

Lead accumulation on the surface of leaves was evaluated. To that end, the above-ground parts of each plant were divided into 2 parts – one part was washed and the other left without washing (Fig. 9). It was demonstrated that the Pb^2+^ concentration in the leaves was 102 mg/kg in the lead-tolerant population and 48 mg/kg in the lead-intolerant population. However, washing removed 40% of lead in the lead-tolerant population and 33% of lead in the lead-intolerant population. This result shows that salt glands are highly effective in removing lead off leaves – particularly in the lead-tolerant population.

### Crystallographic analysis

To find out what exact chemical compounds are forming the crystals on the surface of the leaves of *A. maritima*, we decided to perform a detailed crystallographic analysis. Fortunately, some crystal specimens turned out to be large enough to be measured on a typical single-crystal X-ray diffractometer.

Initial diffraction screening of 430 crystals extracted from *A. maritima* leaves was performed. However, only 130 of them were of high enough quality to establish their unit cell parameters reliably. It turned out that within the analysed set of samples, five different sets of lattice parameters were repeated (Tab. S1, ESI). To establish the exact chemical constitution and the crystal structure of the salts from individual crystals, a full single-crystal X-ray structural analysis was performed for a representative of each type of unit cell.

**Fig. 10:**
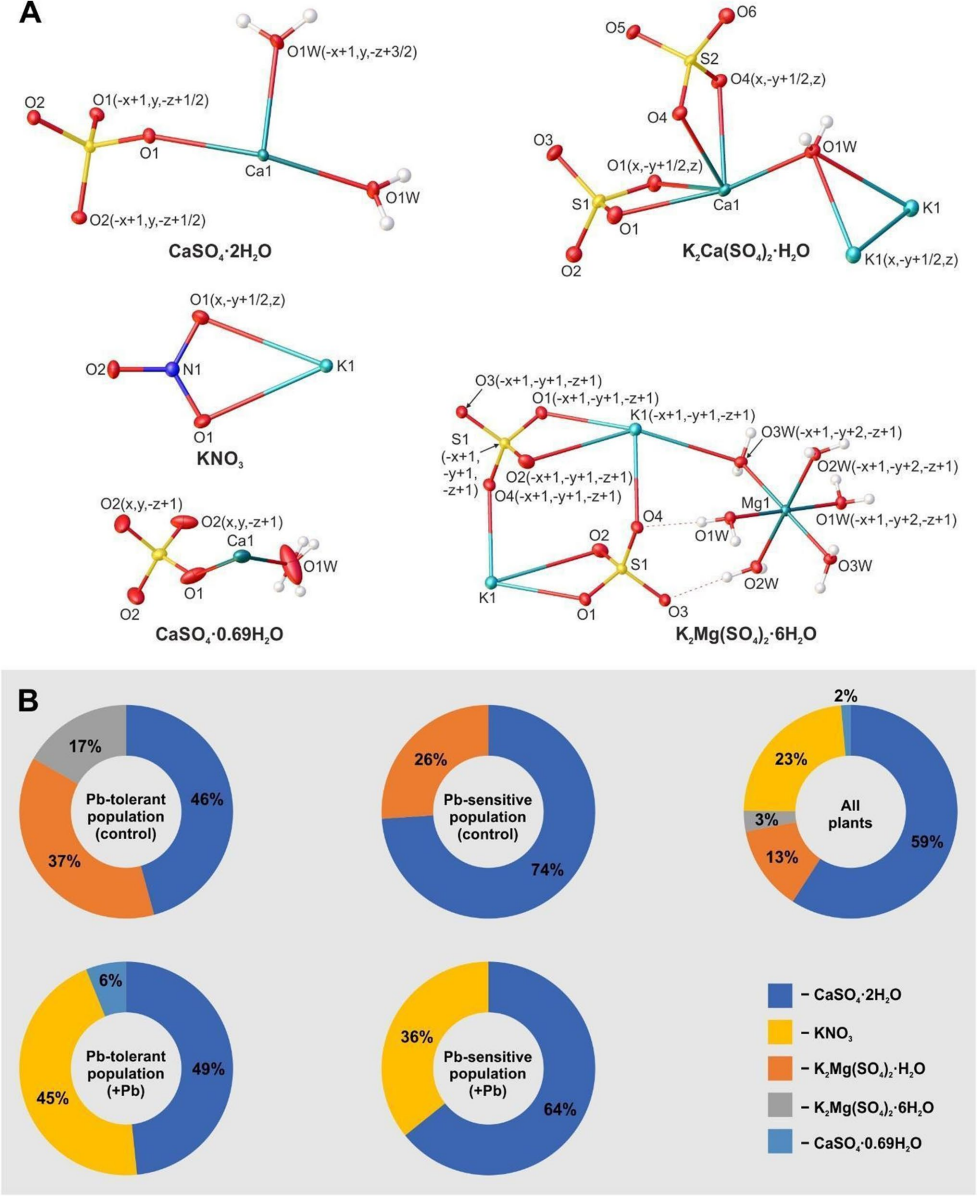
**A** Molecular structure of five inorganic salts forming the crystals on the surface of *A. maritima* leaves. Displacement ellipsoids are drawn at the 50% probability level, while the H-atoms are shown as small spheres of arbitrary radius. Dashed lines represent the hydrogen bonds. **B** Percentage share of crystals of analysed compounds identified on the leaves of *A. maritima* plants from investigated populations

The molecular structure of each compound is shown in Fig. 10A. The details of crystallographic data and the refinement parameters are summarised in Table 1. The complete list of bond lengths, valence and torsion angles can be found in the Supporting Information (Tables S2–S16, ESI). The percentage of crystals of the above-mentioned compounds that could be found in the case of plants from individual batches of their two populations is shown in Fig. 10B. For the plants from the control batches, calcium sulphate dihydrate (46%), potassium calcium sulphate monohydrate (37%) and potassium magnesium sulphate hexahydrate (17%) were found for these belonging to lead-tolerant population, while for the second calcium sulphate dihydrate (74%) was accompanied only by potassium calcium sulphate monohydrate (26%). In turn, for the lead-treated plants, calcium sulphate dihydrate (49%), potassium nitrate (45%) and calcium sulphate subhydrate (6%) were identified within the lead-tolerant population. In contrast, for the lead-sensitive plants, only calcium sulphate dihydrate (64%) and potassium nitrate (36%) were identified.

**Table 1:** Crystal data and structure refinement parameters for the crystals of identified salts from the surface of *A. maritima* leaves

Compound	Calcium sulphate dihydrate (gypsum)	Potassium nitrate	Potassium calcium sulphate monohydrate	Potassium magnesium sulphate hexahydrate	Calcium sulphate subhydrate (CaSO_4_·0.69H_2_O)
Sample	L_Pb_28_1_9-1	B_Pb_17_1_4-2	B_K_4_1_4-4	B_K_6_2_6-1	B_Pb_20_2_6-1
Empirical formula	H4CaO6S	KNO_3_	Ca_0.5_HKO_4.5_S	H_12_K_2_MgO_14_S_2_	H_1.44_CaO_4.72_S
Moiety formula	CaSO_4_·2H_2_O	KNO_3_	K_2_Ca(SO_4_)_2_·H_2_O	K_2_Mg(SO_4_)_2_·6H_2_O	CaSO_4_·0.69H_2_O
Formula weight	172.17	101.11	164.21	402.73	149.11
Temperature/K	100(2)	100(2)	100(2)	100(2)	100(2)
Crystal system	Monoclinic	Orthorhombic	Monoclinic	Monoclinic	Trigonal
Space group	*C*2/*c*	*Pnma*	*P*2_1_/*m*	*P*2_1_/*c*	*P*3_2_21
*a*/Å	6.2562(14)	6.2722(2)	6.2246(4)	6.11490(14)	6.9332(4)
*b*/Å	15.1356(15)	5.39305(16)	7.1218(4)	12.1502(2)	6.9332(4)
*c*/Å	5.6698(10)	9.1392(3)	9.7273(7)	9.0272(2)	6.3460(3)
*α*/°	90	90	90	90	90
*β*/°	114.43(2)	90	104.138(7)	104.507(3)	90
*γ*/°	90	90	90	90	120
Volume/Å^3^	488.83(17)	309.146(17)	418.15(5)	649.31(3)	264.18(3)
*Z*	4	4	4	2	3
*ρ*_calc_g/cm^3^	2.339	2.172	2.608	2.060	2.812
μ/mm^1^	14.808	13.566	20.460	10.662	20.107
*F*(000)	352.0	200.0	328.0	412.0	226.0
Crystal size/mm^3^	0.05 × 0.04 × 0.03	0.17 × 0.13 × 0.11	0.04 × 0.03 × 0.02	0.17 × 0.14 × 0.09	0.03 × 0.02 × 0.02
Radiation	Cu*K*α (*λ* = 1.54184)	Cu*K*α (*λ* = 1.54184)	Cu*K*α (*λ* = 1.54184)	Cu*K*α (*λ* = 1.54184)	Cu*K*α (*λ* = 1.54184)
2*Θ* range for data collection/°	11.694 to 134.106	17.146 to 145.196	9.376 to 133.932	12.476 to 133.958	14.754 to 133.8
Index ranges	− 7 ≤ *h* ≤ 6, − 18 ≤ *k* ≤ 17, − 4 ≤ *l* ≤ 6	− 7 ≤ *h* ≤ 7, − 6 ≤ *k* ≤ 6, − 11 ≤ *l* ≤ 11	− 7 ≤ *h* ≤ 6, − 8 ≤ *k* ≤ 4, − 11 ≤ *l* ≤ 11	− 7 ≤ *h* ≤ 7, − 14 ≤ *k* ≤ 14, − 10 ≤ *l* ≤ 10	− 6 ≤ *h* ≤ 8, − 8 ≤ *k* ≤ 7, − 7 ≤ *l* ≤ 7
Reflections collected	797	3895	1772	9615	1633
Independent reflections	425 [*R*_int_ = 0.0254, *R*_sigma_ = 0.0328]	341 [*R*_int_ = 0.0344, *R*_sigma_ = 0.0153]	813 [*R*_int_ = 0.0226, *R*_sigma_ = 0.0291]	1161 [*R*_int_ = 0.0517, *R*_sigma_ = 0.0255]	323 [*R*_int_ = 0.0248, *R*_sigma_ = 0.0168]
Data/restraints/parameters	425/3/45	341/0/28	813/1/79	1161/9/106	323/3/41
Goodness-of-fit on *F*^2^	1.210	1.175	1.015	1.110	1.148
Final *R* indexes [*I* ≥ 2σ (*I*)]	*R*_1_ = 0.0381, w*R*_2_ = 0.1293	*R*_1_ = 0.0197, w*R*_2_ = 0.0504	*R*_1_ = 0.0287, w*R*_2_ = 0.0715	*R*_1_ = 0.0217, w*R*_2_ = 0.0587	*R*_1_ = 0.0262, w*R*_2_ = 0.0722
Final *R* indexes [all data]	*R*_1_ = 0.0395, w*R*_2_ = 0.1306	*R*_1_ = 0.0204, w*R*_2_ = 0.0510	*R*_1_ = 0.0322, w*R*_2_ = 0.0732	*R*_1_ = 0.0235, w*R*_2_ = 0.0599	*R*_1_ = 0.0283, w*R*_2_ = 0.0736
Largest diff. peak/hole/e Å^−3^	0.53/ − 0.58	0.20/ − 0.33	0.53/ − 0.50	0.32/ − 0.43	0.30/ − 0.31
Flack parameter	–	–	–	–	0.030(14)
CSD refcode	2229824	2229827	2229828	229825	2229826

As for the crystal morphology of analysed salts, all of them differed noticeably in this regard (Fig. 10A). Calcium sulphate dihydrate generally came in the form of thin monoclinic plates mainly gathered in clusters resembling a “desert rose” (a motif often found in its naturally occurring form (gypsum)) (Samy et al. 2012; Watson 1985) or thicker lenticular plates often assembled in druses. Potassium calcium sulphate monohydrate also formed clusters of plates, but in this case, the plates were of a tabular shape. On the other hand, potassium magnesium sulphate hexahydrate, potassium nitrate and subhydrate of calcium sulphate were found as blocks. In the case of the two first compounds, the blocks were relatively well-developed and prismatic, while for the subhydrate, only poorly developed, pseudo prismatic specimens of this type were observed.

Regarding crystals of the same compound within different batches of plants, some distinctions in their appearance can be seen only in the case of calcium sulphate dihydrate. The crystals of this salt appeared to be slightly better formed and larger in the case of plants from the control batch of the lead-sensitive population.

## Discussion

*Armeria maritima* is a halophyte (plant growing on salinised soils, e.g. in seaside areas (Wierzbicka and Słysz 2005; Olko et al. 2008; Abratowska et al. 2015). This species, similar to *Silene vulgaris* (Bringezu et al. 1999), *Dianthus carthusianorum* (Wójcik and Tukiendorf 2014), *Biscutella laevigata* sup. *Wóycicki* (Wierzbicka et al. 2015) or *Arabidopsis arenosa* (Przedpełska and Wierzbicka 2007), exhibits good tolerance for growth and development on zinc–lead waste heaps (e.g. in Bolesław in the Olkusz area, Poland (Abratowska 2013; Wierzbicka and Słysz 2005). The high tolerance of *A. maritima* to heavy metals has already been demonstrated (Abratowska 2006; Żyłkowska 2007; Abratowska et al. 2012). A particularly high tolerance to heavy metals, including lead, was exhibited by the lead-tolerant population of *A. maritima* from a waste heap rich in zinc, lead (1500–2500 mg/kg), cadmium and thallium. Genetic testing showed that *A. maritima* plants growing on waste heaps in Poland are genetically different from the plants growing on non-toxic localities (Abratowska et al. 2012).

### Lead pathway

Our earlier studies, as well as the examinations presented in this paper, allow identification of the Pb^2+^ pathway in *A. maritima* plants. The greatest amounts of lead penetrated into *A. maritima* plants due to the fact that their roots collected lead from the lead-containing ground. This is a typical characteristic of both *A. maritima* and many more plant species (e.g. Szarek–Łukaszewska et al. 2004). In isotope analysis with *Allium cepa*, it was found that lead is collected evenly by the entire surface area of the root. In the radial direction within the root, lead moves relatively fast: at the rate of one cell layer per 5 min (Wierzbicka 1995a). This way the lead reached the stele but radial transport of lead was restricted in the endodermal cell layer. This prevents further apoplastic transport of Pb^2+^ (Casparian strips) (Seregin and Ivanov 1997). Limited Pb^2+^ transport into the deeper layers of the stele occurs thanks to the passage cells within the endoderm. From the roots, some of the lead makes its way to the above-ground parts of the plant, meaning leaf rosettes. Only a few percent of the lead pool collected by a plant gets to the above-ground part of the plant. Retaining the highest amounts of lead in the roots and its limited translocation into the leaves allows protection of above-ground plants – mostly the photosynthetic apparatus – against the toxic action of lead.

Lead went from the roots to the leaves via vascular bundles. The highest amounts of lead were found in vascular bundle cells (in the phloem). From the vascular bundles, lead penetrated successive layers of leaf mesophyll and reached the epidermis. This indicates that lead concentration in the mesophyll cells of the leaf depended on distance from the vascular bundle. The process is relatively slow as lead was shown to only penetrate around 5 mesophyll cell layers after 24 h of incubation – one cell layer per 4.8 h. The salt glands secreted a multi-ionic salt solution with lead-containing salts which crystallised on the surface of leaves.

### High tolerance of *A. maritima* to lead

A. The characteristic features of the structure of the *A. maritima* leaf are extensive intercellular spaces in the mesophyll. This is where Pb^2+^ accumulated in the highest amounts—which was demonstrated both through the visualisation of lead with dithizone histochemistry and through X-ray microanalysis with an electron microscope. Such a lead pool will not be toxic for the cells.
B. In cells, lead deposits were found in cell walls, cytoplasm and chloroplasts. Our study into *A. maritima* showed that the metabolism of those cells changed. Glutathione (GSH) contents increased; glutathione is an antioxidant involved in anti-free radical protection mechanisms. In addition, malic acid levels went up (Olko et al. 2008).
C. Removal of lead onto the leaf surface leads to significant lead detoxification. The presence of salt glands on leaves is a characteristic feature of most halophytes. This study determined in detail what elements, and in what amounts, were secreted by salt glands during a 6-month growth. It was shown that the glands expelled multi-ingredient salt solutions, which crystallised on the leaf. They consisted mostly of lead, sulphur and calcium and, in smaller amounts, phosphorus, magnesium, chlorine and potassium. All the detected elements were present as ions in the mineral medium in which the roots were submersed. Phosphorus was the exception: it was not found in the medium as it precipitates as Pb_3_(PO_4_)_2_. Therefore, it seems that the phosphorus found in the crystals was of endogenous origin.

From among all analysed samples, the lead-tolerant population was the most effective in salt expulsion. The plants in the *A. maritima* lead-tolerant population expelled 40% of lead accumulated by their leaves within one vegetation period. It needs to be stressed that the transport of lead from the roots to the leaves was twice as high in the lead-tolerant population compared to the lead-sensitive one. These are important characteristics which predispose *A. maritima* plants, the lead-tolerant populations in particular, for the phytoremediation of lead-contaminated soils. In our opinion, the above manner of protection against lead is exceptionally high and effective.

In other studies into halophytes, Na^+^, Cl^−^ and Ca^2+^ ions were most frequently detected in salt gland secretions. However, the composition depended on the species of the plant, composition of the ground and the employed analytical method. For this reason, it is difficult to compare the results of this paper with other studies.

However, the value of our study can be found in the evaluation of the work of salt glands throughout the entire vegetation period of *A. maritima* plants. Demonstration that lead is expelled in this manner was particularly significant. During our experiments, lead was provided only through the roots of plants and there was no possibility whatsoever to interfere with the composition of the crystals produced on the leaves. Under natural conditions, this is not possible because of weather (e.g. occurrence of rains and drew) or particulate matter of varied chemical composition falling onto the leaves.

#### Structure of crystals

In the course of our crystallographic studies, the presence of crystals of five different inorganic salts on the surface of *A. maritima* leaves was revealed. A comparison of the crystal structure of investigated compounds with these already reported shows that almost all systems presented in this paper are already known (Boeyens et al. 2002; Nimmo and Lucas 1976; Corazza and Sabelli 1967; Kannan and Viswamitras 1965). These five compounds occur in the Earth’s crust in the form of minerals: gypsum (calcium sulphate dihydrate) (Wooster 1936; Schofield et al. 1996; Comodi et al. 2008), niter (potassium nitrate) (Palache et al. 1951), syngenite (potassium calcium sulphate monohydrate) (Corazza and Sabelli 1967; Palache et al. 1951) and picromerite (potassium magnesium sulphate hexahydrate) (Bosi et al. 2009). However, this does not depreciate the value of our discovery because almost all inorganic salts found on *A. maritima* leaves have never been associated with the crystallisation processes induced or influenced by non-human organisms with the exception of gypsum. Even though gypsum is the dominant terrestrial sulphate present in a wide range of environments, evidence of its production by plants is still sparse (Storey and Thompson 1994; Pritchard et al. 2000; He et al. 2012; Van Driessche et al. 2019). Knowledge that the potential function these crystals could fulfil is also limited (He et al. 2012; Van Driessche et al. 2019). Additionally, to the best of our knowledge, the crystal structure of calcium sulphate subhydrate (CaSO_4_·0.69H_2_O) is reported for the first time although several solvates of calcium sulphate with the formula CaSO_4_·*n*H_2_O, where 0.5 ≤ *n* ≤ 0.8, are known (Christensen et al. 2008; Bezou et al. 1995; Schmidt et al. 2011).

However, such systems are usually formed by the dehydration of gypsum at high temperatures. Moreover, in most cases, their existence was proven by powder diffraction methods, and there is still a debate on their construction and phase transformations. The lattice constraints of the subhydrate investigated by us are the closest to those reported for the *β*-hemihydrate of CaSO_4_, which also crystallises in the trigonal space group *P*3_2_21, but in this system, the water content relative to the CaSO_4_ unit is different (Abriel and Nesper 1993).

Comparing the percentage share of crystals of the investigated compounds that could be found in the case of plants from individual batches (Fig. 10), it can be seen that gypsum was significant in all the investigated plant populations. From a chemical point of view, this is not surprising as Ca^2+^ and SO_4_^2−^ ions comprise a significant share of the Knop medium used to feed the plants, and gypsum is the most stable form of calcium sulphate arising during its precipitation from aqueous solution at low temperatures (< 40–60 °C) (Van Driessche et al. 2019). However, this is notable in the context of an understanding a crystallisation processes induced by plants. Taking into account the shares of individual compounds identified for respective populations of plants, it can be seen that those plants can indeed differ in their ability to produce various crystalline salts. Research results show that the diversity of inorganic salts creating crystals on leaves is noticeably greater in the case of plants from the lead-tolerant population. Intriguingly, salts containing two metal cations were found only on the leaves of plants from the control batch (both populations), while for representatives of both populations, which were additionally treated with lead ions, only crystals of simple salts were present. The above observations are intriguing, but it is difficult to explain them unambiguously. Perhaps the phenomenon of greater crystalline salt diversity in the case of plants from the lead-tolerant population was related to the fact that the multi-component solutes transported outside the leaf through salts glands were more concentrated. Such a hypothesis seems to be confirmed by the results obtained from X-ray microanalysis, where higher concentrations of individual elements were generally recorded for plants belonging to this specific population. The higher ion concentration idea may also fit the possibility of the appearance of the metastable subhydrate of calcium sulphate. As mentioned in the previous paragraph, systems of this type are generally formed by the partial dehydration of gypsum at high temperatures (Christensen et al. 2008; Bezou et al. 1995; Schmidt et al. 2011). However, some literature reports suggest that the hemihydrate can be formed from gypsum in solutions of lower water activity, such as MgCl_2_ or concentrated strong acids near room temperature (Freyer and Voigt 2003). Lead ions may also somehow interfere with the formation of multi-component crystalline salts. However, during this study, we could not verify this hypothesis. Unfortunately, knowledge about the formation of double salts with alkali and alkaline earth sulphates is still incomplete even under strictly controlled laboratory conditions (Freyer and Voigt 2003). Needless to say that biological systems introduce many more variables.

No lead ions were observed within the crystal network of any crystal-forming compounds during the performed structural studies. However, it does not contradict the results obtained using EDX, where the presence of lead was noted. There are literature reports that some metal ions co-precipitated with the Ca^2+^ and SO_4_^2−^ ions can be absorbed on the surface of arising gypsum (Hamdon and Al Hadad 2007). Additionally, it is known that gypsum can effectively remove the Pb^2+^ ions from highly contaminated aqueous solutions by the growth of an epitactic layer of the lead sulphate on its surface (Astilleros et al. 2010). As mentioned earlier, gypsum was the most frequently noted crystalline phase on analysed *A. maritima* leaves. Interestingly, electron microscopy images taken by us show that some of the investigated crystals (this applies not only to gypsum) were covered by some glaze. It does not look like an epitaxial layer, but it might be an amorphous multi-component enamel containing, among others, the Pb^2+^ ions. This type of material could also be seen in some places directly on the surface of the leaves. Of course, it cannot be excluded that a thin epitaxial film of lead sulphate was formed on the gypsum crystals or that the examined leaves contained crystals entirely made of lead salts, but their size and quality prevented from analysing them using a home single-crystal diffractometer. Interestingly, the specific internal structure of gypsum might also enable the creation of solid solutions incorporating bivalent metal cations. Recently, the formation of such systems containing small amounts of Cu^2+^, Zn^2+^ and Cd^2+^ ions was reported (Ma et al. 2020). The explanation of this phenomenon was proposed based on the results of DFT calculations. Theoretical studies indicated that the above ions might be built into the crystal lattice of gypsum in two different ways. The Cd^2+^ and Zn^2+^ might replace the Ca^2+^ ions by their isomorphic substitution, whereas the Cu^2+^ cations might be incorporated into the gypsum’s water channels. It is unknown if gypsum can form similar systems containing lead. However, reports on hydroxyapatite showed that substituting calcium with Pb^2+^ cations was possible under certain conditions (Bigi et al. 1991; Ellis et al. 2006). Pb^2+^ ion concentrations were too low to detect within the structure of the gypsum crystals through X-ray structure determination. It is worth noting that the subhydrate crystals also contained similar spaces filled with molecules of solvent (Fig. S1, ESI).

Differences in the morphology of gypsum crystals within different batches of plants may have resulted from lower concentrations of ions secreted by the salt glands, which made the crystallisation process a bit slower, and the lack of Pb^2+^ ions in the nutrient solution, which could settle on the crystal surface and adversely affect their growth. Unfortunately, at this stage, the available data allowed only speculation on this matter.

## Conclusions

The mechanisms resulting in an exceptionally high tolerance of *A. maritima* plants for heavy metals (lead) include:

- The possibility to accumulate lead in large intercellular spaces.
- Accumulation of lead in the cell walls of vascular bundles, strengthening cells (sclerenchyma), mesophyll cells, epidermal cells and the epidermal hairs.
- Lead expulsion by the salt glands. The lead-tolerant population transported twice as much lead from the roots to the leaves compared to the lead-sensitive population. As much as 40% of the lead pool in the leaf tissues was expelled by the salt glands onto the surface of the leaves.

These characteristics indicate the high phytoremediation capabilities of the *A. maritima* (halophyte) plants, particularly of the lead-tolerant population found growing in Poland.

Furthermore, it was the first time the structure of these crystals was studied. The endeavours can be summarised as follows:

- Examination of the concentration of individual elements via the X-ray microanalysis proved that the presence of lead ions could generally be detected on the surface of the crystalline material appearing on the leaves. This element was most often found in the company of calcium and sulphur.
- Crystallographic studies, allowed us to determine the identity and crystal structure of the compounds that form them. They were calcium sulphate dihydrate (gypsum), potassium calcium sulphate monohydrate (syngenite), potassium magnesium sulphate hexahydrate (picromerite) and calcium sulphate subhydrate CaSO_4_·0.69H2O. With the exception of gypsum, none of the above compounds have been reported so far in the context of bio-influenced crystallisation processes.
- Pb^2+^ cations have not been found within any crystal network from the analysed set; however, this does not rule out their presence. According to the literature reports, gypsum (the dominant crystalline phase present on the leaves of *A. maritima* plants from all analysed populations) has the ability to absorb metal ions on its surface and accumulate some of them inside. In the latter case, the ions can be located within specific water channels present in its structure or can replace calcium ions on the basis of isomorphic substitution.
- Interestingly, the studied plant populations differ regarding the crystals they produce. The research results show that a broader palette of chemical compounds was found on the surface of the leaves of plants from the lead-tolerant population.
- Results indicate that the presence of lead ions may affect the crystalline phases formed.

### Supplementary Information

Below is the link to the electronic supplementary material.

Supplementary file1 (DOCX 4.92 MB)

## Data Availability

The CCDC 2229824 and 2229828 contain the supplementary crystallographic data for this paper. The data can be obtained free of charge from the Cambridge Crystallographic Data Centre via www.ccdc.cam.ac.uk/structures.
