# Supplementary material for: Role of the salt glands of Armeria maritima (halophyte) in removal of lead from tissues
Source: Environ Sci Pollut Res Int. 2024 May 24;31(25):37790–809. doi: 10.1007/s11356-024-33624-z (PMC11182854; doi:10.1007/s11356-024-33624-z)
Supplement: Supplementary file 1 — Supplementary file1 (DOCX 4.92 MB) [file 11356_2024_33624_MOESM1_ESM.docx]

**Electronic Supporting Information (ESI)**

**
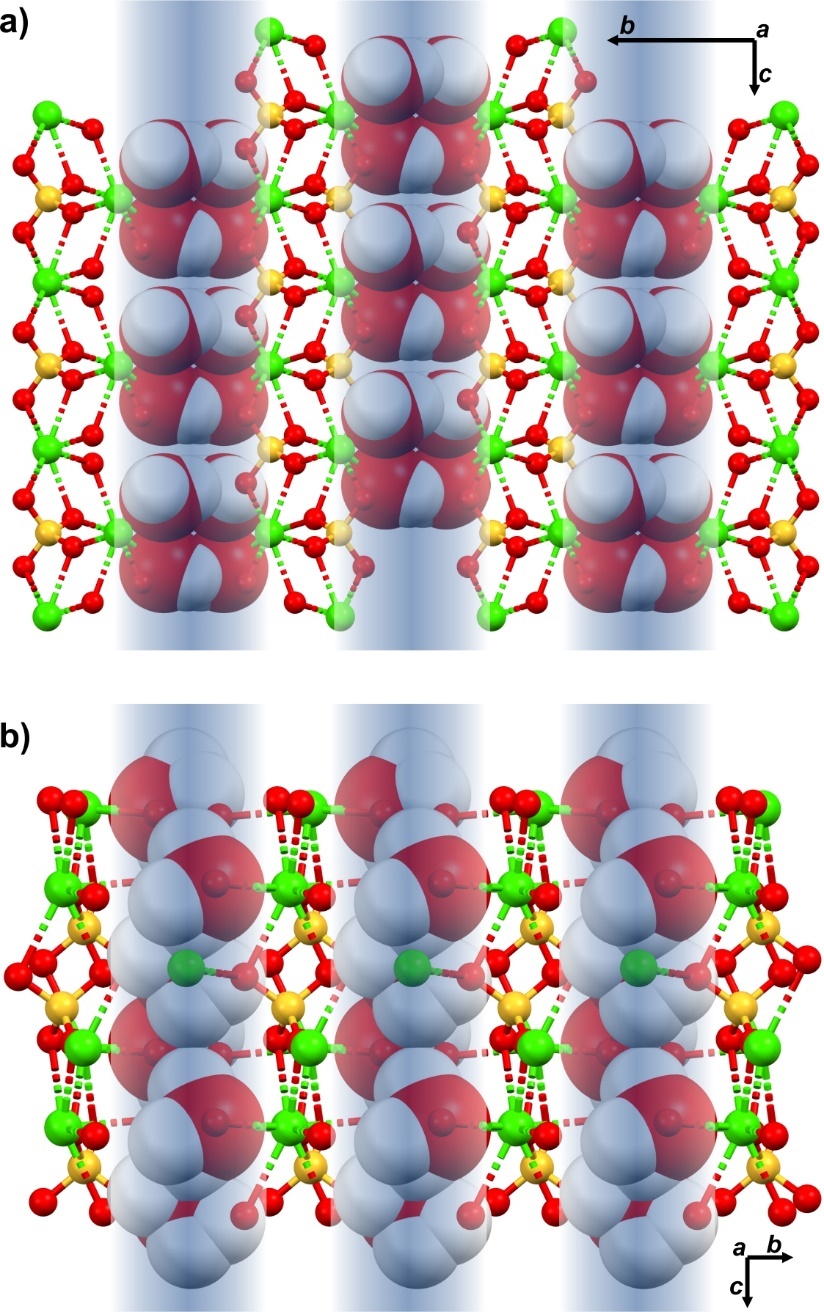
**

**Figure S1.** Packing of the water molecules in the channels formed by the CaO_8_ units in the crystal network of gypsum (a), and subhydrate of calcium sulfate CaSO_4_·0.69H_2_O (b). Water channels are highlighted in blue.

**Table S1.** Unit cell parameters of investigated crystals selected from the surface of *A. maritima* leaves and the SEM images illustrating their morphology.

| **Population** | **Sample** | **Type of experiment** | **Bravais lattice type** | **Unit cell parameters**  ***a*, *b*, *c*, *α*, *β*, *γ* [Å,°]** | **Compound** | **An image of the spot from the crystal was selected.** |
| --- | --- | --- | --- | --- | --- | --- |
| Metal tolerant (control) | B_K_1_1_1-1 | pre | mC | 6.29(6)  15.16(14)  5.65(7)  90.4(9)  114(1)  89.7(7) | Gypsum | 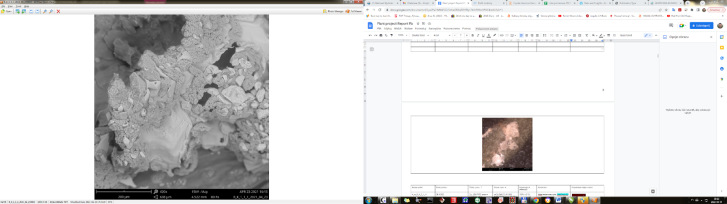 |
| Metal tolerant (control) | B_K_2_1_1-1 | pre | mC | 6.266(17)  15.16(5)  5.679(10)  90.0  114.3(3)  90.0 | Gypsum | 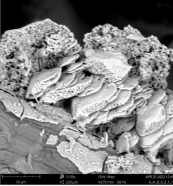 |
| Metal tolerant (control) | B_K_2_1_1-2 | pre | mC | 6.266(15)  15.25(3)  5.66(3)  90.0  114.4(4)  90.0 | Gypsum |  |
| Metal tolerant (control) | B_K_2_1_1-3 | pre | mC | 6.29(2)  15.11(3)  5.695(15)  90.0  114.2(4)  90.0 | Gypsum |  |
| Metal tolerant (control) | B_K_4_1_4-3 | pre | mP | 6.205(13)  7.237(9)  9.42(2)  90.0  102.5(3)  90.0 | Syngenite | 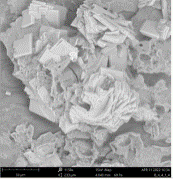 |
| Metal tolerant (control) | B_K_4_1_4-4 | full | mP | 6.2246(4)  7.1218(4)  9.7273(7)  90  104.138(7)  90 | Syngenite |  |
| Metal tolerant (control) | B_K_4_1_8-3 | pre | mP | 6.2(2)  7.1(5)  9.72(10)  90(2)  104(2)  90(4) | Syngenite | 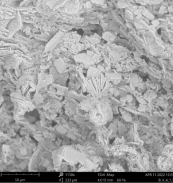 |
| Metal tolerant (control) | B_K_5_1_6-2 | Pre | mC | 6.24(6)  15.3(3)  5.65(3)  90.0  114.2(9)  90.0 | Gypsum | 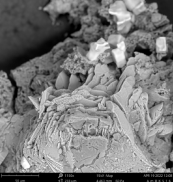 |
| Metal tolerant (control) | B_K_5_1_6-3 | Full | mC | 6.265(2)  15.138(3)  5.6774(14)  90  114.35(4)  90 | Gypsum |  |
| Metal tolerant (control) | B_K_5_1_8-1 | pre | mP | 6.25(5)  7.18(4)  9.73(8)  87.7(6)  102.8(7)  89.9(6) | Syngenite | 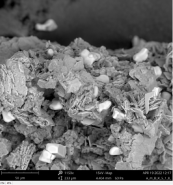 |
| Metal tolerant (control) | B_K_5_1_8-4 | pre | mP | 6.249(6)  7.135(7)  9.778(10)  90.0  103.97(10)  90.0 | Syngenite |  |
| Metal tolerant (control) | B_K_5_1_8-6 | pre | mP | 6.219(13)  7.083(18)  9.714(17)  89.8(3)  104.5(3)  90.3(4) | Syngenite |  |
| Metal tolerant (control) | B_K_5_1_8-7 | pre | mP | 6.220(7)  7.144(9)  9.725(12)  90.0  104.25(13)  90.0 | Syngenite |  |
| Metal tolerant (control) | B_K_5_1_10-2 | pre | mC | 6.250(2)  15.110(9)  5.6600(18)  90.00(4)  114.39(4)  90.00(4) | Gypsum | 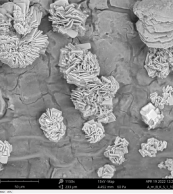 |
| Metal tolerant (control) | B_K_6_1_9-1 | pre | mC | 6.30(8)  15.3(2)  5.72(6)  89(1)  113(1)  89(1) | Gypsum | 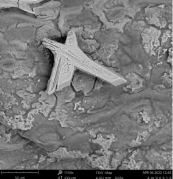 |
| Metal tolerant (control) | B_K_6_1_9-2 | pre | mC | 6.27(3)  15.12(6)  5.66(3)  89.8(4)  114.0(4)  89.9(4) | Gypsum |  |
| Metal tolerant (control) | B_K_6_2_6-1 | full | mP | 6.11490(14)  12.1502(2)  9.0272(2)  90  104.507(3)  90 | Picromerite | 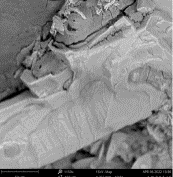 |
| Metal tolerant (control) | B_K_6_2_6-2 | pre | mP | 6.101(13)  12.123(16)  9.002(16)  89.85(19)  104.3(2)  89.94(15) | Picromerite |  |
| Metal tolerant (control) | B_K_6_2_6-3 | pre | mP | 6.14(2)  12.17(4)  9.03(3)  90.2(3)  104.9(3)  90.4(3) | Picromerite |  |
| Metal tolerant (control) | B_K_6_2_13-1 | pre | mP | 6.10(2)  12.116(6)  9.001(19)  89.8(2)  104.3(3)  90.13(15) | Picromerite | 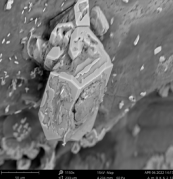 |
| Metal tolerant (control) | B_K_8_1_2-1 | pre | mC | 6.3(2)  15.07(14)  5.6(2)  89(3)  116(6)  89(3) | Gypsum | 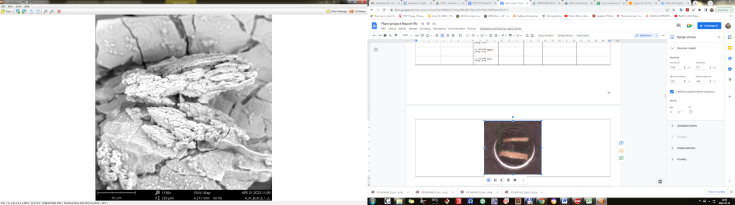 |
| Metal tolerant (control) | B_K_8_1_2-2 | pre | mC | 6.260(5)  15.125(6)  5.668(4)  90.0  114.37(8)  90.0 | Gypsum |  |
| Metal tolerant (control) | B_K_8_2_4-1 | pre | mP | 6.202(9)  7.079(12)  9.71(2)  90.0  104.1(2)  90.0 | Syngenite | 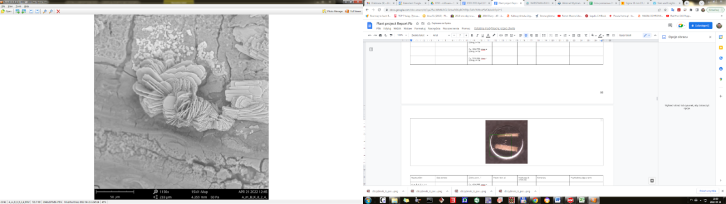 |
| Metal tolerant (control) | B_K_8_2_9-3 | pre | mP | 6.38(3)  7.29(5)  9.7(2)  91.0(8)  105(1)  90.0(5) | Syngenite | 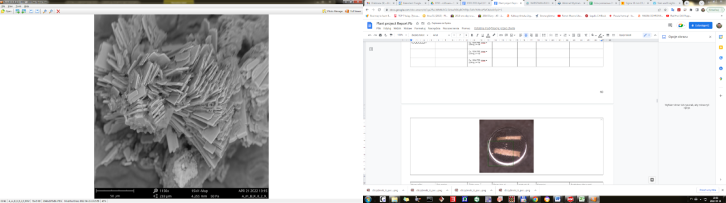 |
| Metal intolerant (control) | L_K_10_1_2-1 | full | mC | 6.2598(5)  15.1329(8)  5.6732(5)  90  114.335(10)  90 | Gypsum | 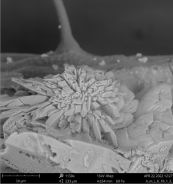 |
| Metal intolerant (control) | L_K_10_1_2-3 | pre | mC | 6.24(15)  15.11(12)  5.66(5)  90.3(7)  114.6(8)  89.8(7) | Gypsum |  |
| Metal intolerant (control) | L_K_10_1_4-1 | pre | mC | 6.28(4)  15.16(3)  5.69(2)  90.0  114.6(5)  90.0 | Gypsum | 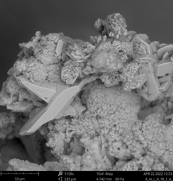 |
| Metal intolerant (control) | L_K_10_1_4-2 | pre | mC | 6.29(5)  14.99(7)  5.69(6)  91.2(6)  114.5(9)  88.6(5) | Gypsum |  |
| Metal intolerant (control) | L_K_10_1_4-3 | pre | mC | 6.25(12)  15.1(2)  5.66(5)  90(1)  114(1)  90(2) | Gypsum |  |
| Metal intolerant (control) | L_K_10_1_5-1 | pre | mC | 6.289(19)  15.12(8)  5.684(14)  89.8(3)  114.2(3)  90.1(3) | Gypsum | 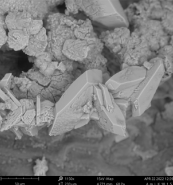 |
| Metal intolerant (control) | L_K_10_1_5-3 | pre | mC | 6.23(10)  14.9(3)  5.66(10)  90.8(10)  114(2)  89(1) | Gypsum |  |
| Metal intolerant (control) | L_K_10_2_5-1 | pre | mC | 6.25(2)  15.16(6)  5.71(3)  89.8(4)  114.4(4)  90.1(3) | Gypsum | 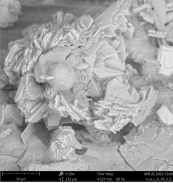 |
| Metal intolerant (control) | L_K_10_2_9-1 | pre | mP | 6.23(3)  7.18(6)  9.54(7)  90.4(6)  104.2(5)  90.2(5) | Syngenite | 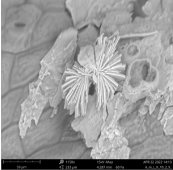 |
| Metal intolerant (control) | L_K_11_1_5-3 | pre | mC | 6.25(7)  15.30(14)  5.68(10)  90.0  115(2)  90.0 | Gypsum | 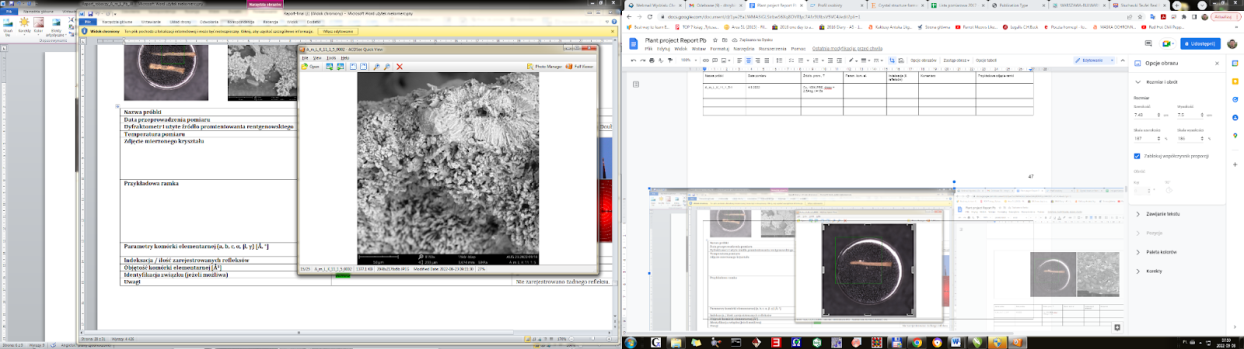 |
| Metal intolerant (control) | L_K_12_1_1-1 | pre | mC | 6.31(7)  15.17(7)  5.71(3)  90.0  114.4(9)  90.0 | Gypsum | 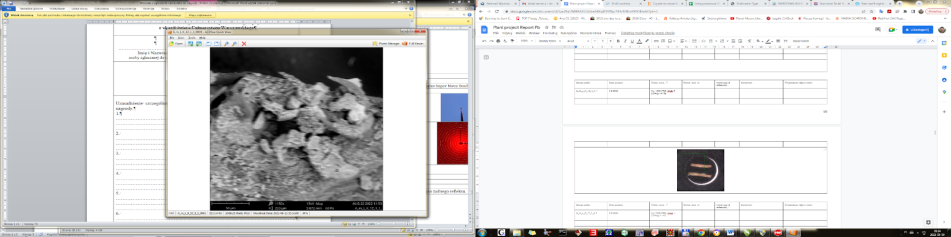 |
| Metal intolerant (control) | L_K_12_1_8-1 | pre | mC | 6.306(18)  15.08(8)  5.72(3)  90.0  114.5(5)  90.0 | Gypsum | 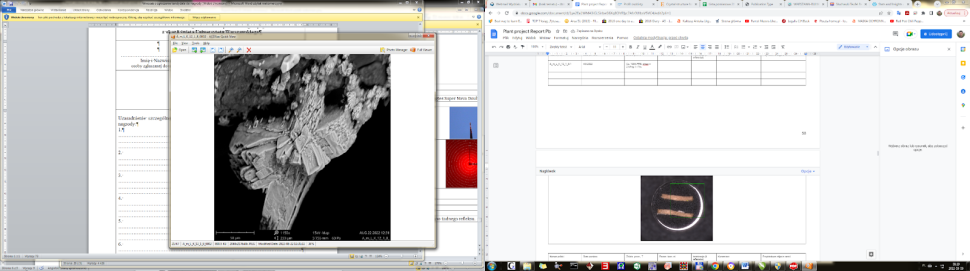 |
| Metal intolerant (control) | L_K_12_1_8-2 | pre | mC | 6.26(7)  15.19(7)  5.65(4)  90.1(5)  113.9(9)  91.0(7) | Gypsum |  |
| Metal intolerant (control) | L_K_12_1_8-3 | pre | mC | 6.26(2)  15.172(15)  5.669(16)  90.0  114.2(3)  90.0 | Gypsum |  |
| Metal intolerant (control) | L_K_12_2_2-2 | pre | mP | 6.26(2)  7.12(2)  9.71(2)  90.0  105.0(6)  90.0 | Syngenite | 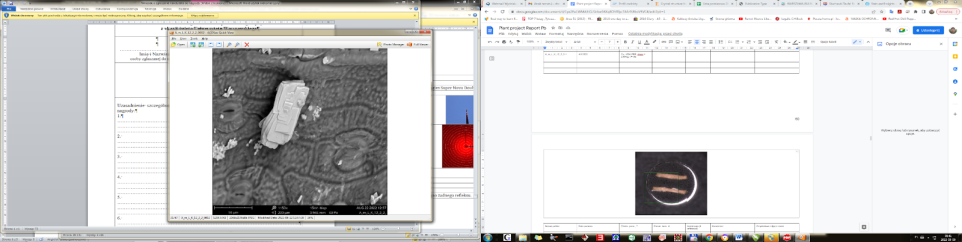 |
| Metal intolerant (control) | L_K_13_2_6-1 | pre | mC | 6.27(2)  15.12(5)  5.675(13)  90.0  114.4(3)  90.0 | Gypsum | 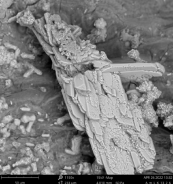 |
| Metal intolerant (control) | L_K_13_2_7-1 | pre | mC | 6.27(4)  15.11(9)  5.70(3)  90.0  114.3(7)  90.0 | Gypsum | 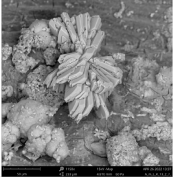 |
| Metal intolerant (control) | L_K_14_1_9-1 | pre | mC | 6.257(9)  15.126(16)  5.674(10)  90.0  114.30(18)  90.0 | Gypsum | 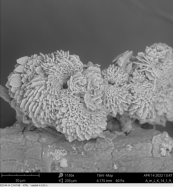 |
| Metal intolerant (control) | L_K_14_1_9-3 | pre | mC | 6.16(9)  15.12(15)  5.88(11)  91(1)  114(2)  90(1) | Gypsum |  |
| Metal intolerant (control) | L_K_14_1_10-1 | pre | mC | 6.26(2)  15.14(5)  5.69(2)  89.9(3)  114.6(4)  90.0(3) | Gypsum | 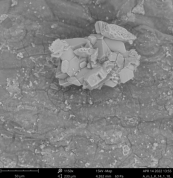 |
| Metal intolerant (control) | L_K_14_1_10-2 | pre | mC | 6.26(2)  15.14(5)  5.69(2)  89.9(3)  114.6(4)  90.0(3) | Gypsum |  |
| Metal intolerant (control) | L_K_14_2_6+7-3 | pre | mC | 6.24(6)  15.15(3)  5.70(3)  90.0  114.2(7)  90.0 | Gypsum | 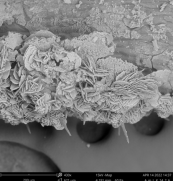 |
| Metal intolerant (control) | L_K_14_2_9-1 | pre | mC | 6.23(6)  15.05(6)  5.646(18)  90.0  114.3(7)  90.0 | Gypsum | 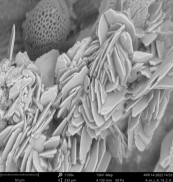 |
| Metal intolerant (control) | L_K_15_1_12-2 | pre | mP | 6.241(7)  7.097(14)  9.728(15)  90.0  104.9(3)  90.0 | Syngenite | 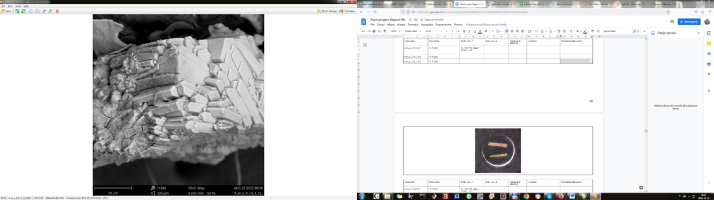 |
| Metal intolerant (control) | L_K_15_1_12-3 | pre | mP | 6.218(8)  7.100(14)  9.73(2)  90.0  104.48(14)  90.0 | Syngenite |  |
| Metal intolerant (control) | L_K_15_1_13-1 | pre | mP | 6.225(12)  7.11(2)  9.72(3)  90.0  104.14(19)  90.0 | Syngenite | 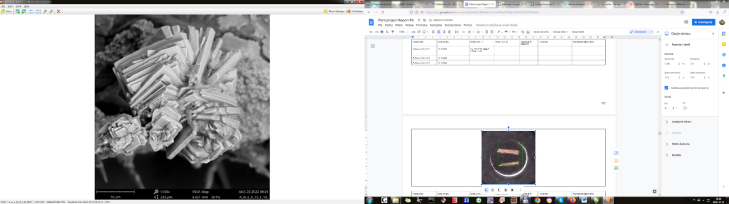 |
| Metal intolerant (control) | L_K_15_1_13-2 | pre | mP | 6.20(2)  7.115(15)  9.72(2)  90.0  104.0(3)  90.0 | Syngenite |  |
| Metal intolerant (control) | L_K_15_1_15-1 | pre | mP | 6.219(10)  7.120(17)  9.71(6)  90.0  103.9(3)  90.0 | Syngenite | 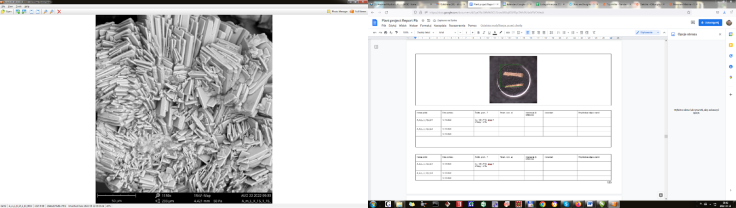 |
| Metal intolerant (control) | L_K_15_1_15-2 | pre | mP | 6.22(4)  7.11(2)  9.73(2)  90.0  104.6(3)  90.0 | Syngenite |  |
| Metal intolerant (control) | L_K_16_1_3-3 | pre | mC | 6.29(8)  15.18(17)  5.69(5)  89.9(8)  114.6(10)  88.2(9) | Gypsum | 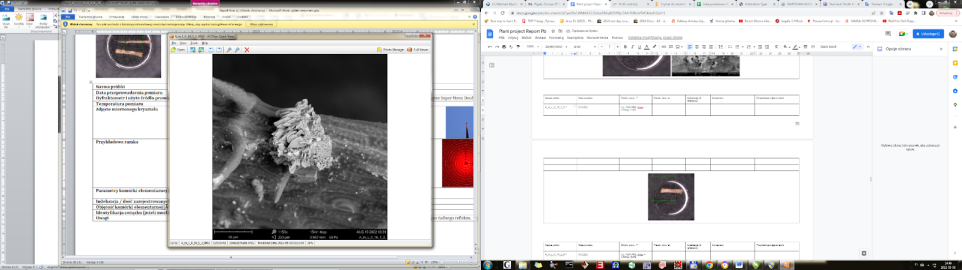 |
| Metal intolerant (control) | L_K_16_1_9-1 | pre | mC | 6.29(18)  15.09(6)  5.67(10)  90.0  115(3)  90.0 | Gypsum | 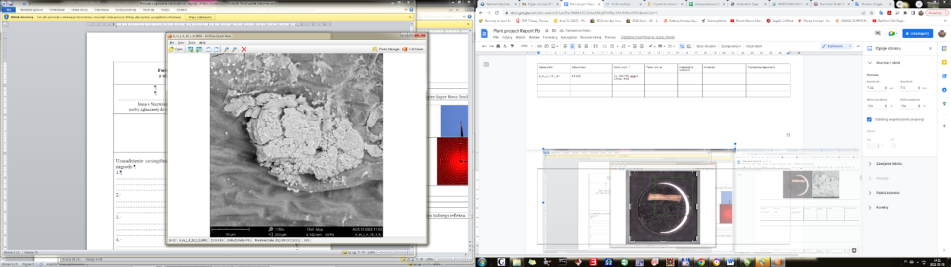 |
| Metal tolerant (+ Pb) | B_Pb_17_2-1 | pre | oP | 6.48(2)  5.391(10)  9.24(2)  90.0  90.0  90.0 | Niter | 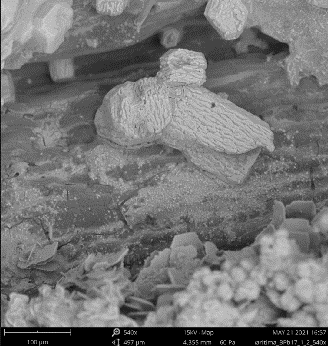 |
| Metal tolerant (+ Pb) | B_Pb_17_2-2 | pre | oP | 6.425(14)  5.425(12)  9.20(2)  89.70(18)  89.90(18)  89.92(18) | Niter |  |
| Metal tolerant (+ Pb) | B_Pb_17_2-3 | pre | oP | 6.422(8)  5.413(5)  9.165(12)  90.0 90.0  90.0  318.6(6) | Niter |  |
| Metal tolerant (+ Pb) | B_Pb_17_1_6-1 | pre | oP | 6.44(2)  5.400(8)  9.206(14)  90.0  90.0  90.0 | Niter | 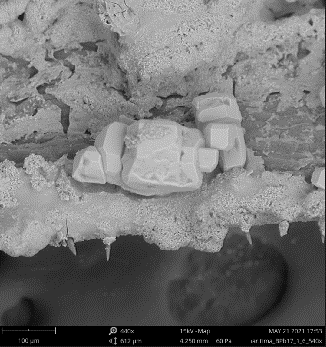 |
| Metal tolerant (+ Pb) | B_Pb_17_1_6-2 | pre | oP | 6.426(4)  5.4197(14)  9.169(3)  90.0  90.0  90.0 | Niter |  |
| Metal tolerant (+ Pb) | B_Pb_17_1_6-3 | pre | oP | 6.420(7)  5.416(5)  9.159(3)  90.0  90.0  90.0 | Niter |  |
| Metal tolerant (+ Pb) | B_Pb_17_1_6-4 | pre | oP | 6.428(3)  5.422(3)  9.169(3)  90.0  90.0  90.0 | Niter |  |
| Metal tolerant (+ Pb) | B_Pb_17_1_6-5 | pre | oP | 6.441(2)  5.4278(18)  9.150(11)  90.0  90.0  90.0 | Niter |  |
| Metal tolerant (+ Pb) | B_Pb_17_1_4_1 | pre | oP | 6.425(6)  5.417(5)  9.164(5)  90.0  90.0  90.0 | Niter | 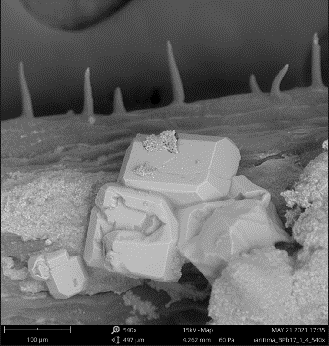 |
| Metal tolerant (+ Pb) | B_Pb_17_1_4-2 | full | oP | 6.27222)  5.39305(16)  9.1392(3)  90  90  90 | Niter |  |
| Metal tolerant (+ Pb) | B_Pb_17_1_4-3 | pre | oP | 6.43(2)  5.397(9)  9.194(12)  90.0  90.0  90.0 | Niter |  |
| Metal tolerant (+ Pb) | B_Pb_20_2_6-1 | full | hP | 6.9332(4)  6.9332(4)  6.3460(4)  90  90  120 | CaSO_4_·0.69H_2_O | 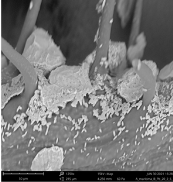 |
| Metal tolerant (+ Pb) | B_Pb_20_2_6-2 | pre | hP | 6.934(16)  6.934(16)  6.35(2)  90.0  90.0  120.0 | CaSO_4_·0.69H_2_O |  |
| Metal tolerant (+ Pb) | B_Pb_21_1_2-2 | pre | mC | 6.270(12)  15.22(3)  5.682(15)  90.09(18)  114.4(2)  89.74(17) | Gypsum | 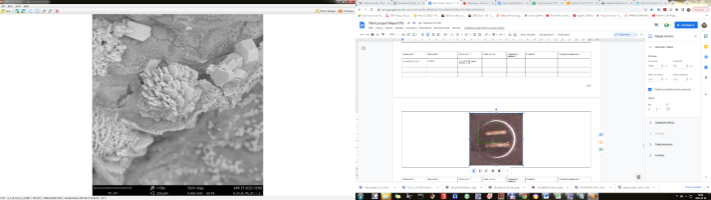 |
| Metal tolerant (+ Pb) | B_Pb_21_1_2-3 | pre | mC | 6.31(12)  15.13(10)  5.67(17)  90.0  115(4)  90.0 | Gypsum |  |
| Metal tolerant (+ Pb) | B_Pb_21_1_3-1 | pre | mC | 6.34(4)  15.26(2)  5.71(3)  90.0  114.6(7)  90.0 | Gypsum | 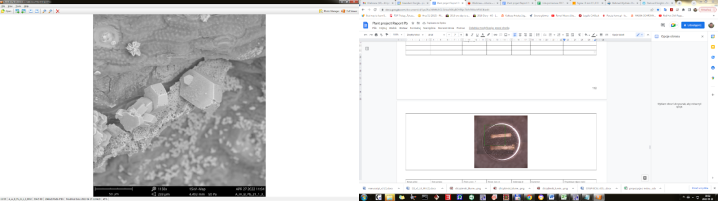 |
| Metal tolerant (+ Pb) | B_Pb_21_1_9-1 | pre | mC | 6.272(17)  15.21(5)  5.70(4)  90.0  114.8(6)  90.0 | Gypsum | 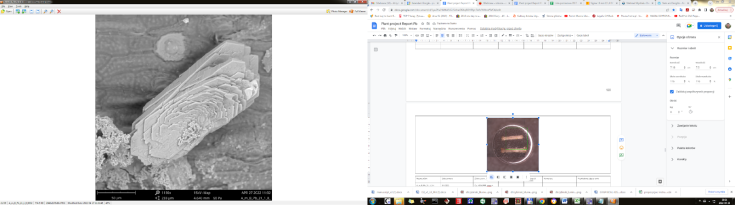 |
| Metal tolerant (+ Pb) | B_Pb_21_1_9-2 | pre | mC | 6.216(15)  15.13(3)  5.660(16)  90.0  113.9(4)  90.0 | Gypsum |  |
| Metal tolerant (+ Pb) | B_Pb_21_1_9-3 | pre | mC | 6.28(6)  14.97(17)  5.68(4)  90.0  114(1)  90.0 | Gypsum |  |
| Metal tolerant (+ Pb) | B_Pb_21_1_10-1 | pre | mC | 6.32(4)  15.26(4)  5.67(7)  90.0  115(1)  90.0 | Gypsum | 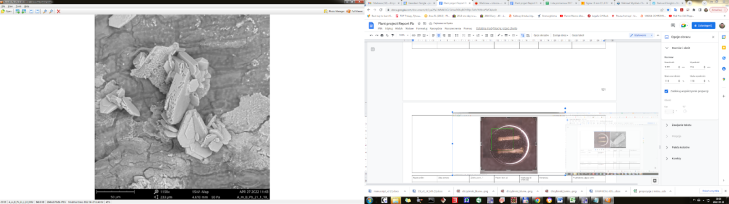 |
| Metal tolerant (+ Pb) | B_Pb_21_1_10-2 | pre | mC | 6.1(7)  15.4(3)  5.5(5)  90.0  112(14)  90.0 | Gypsum |  |
| Metal tolerant (+ Pb) | B_Pb_22_1_7-1 | pre | mC | 6.31(6)  15.23(5)  5.67(5)  90.0  114(1)  90.0 | Gypsum | 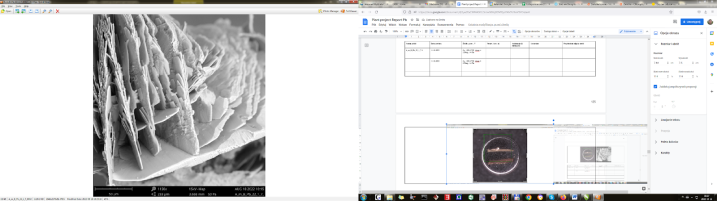 |
| Metal tolerant (+ Pb) | B_Pb_22_1_7-2 | pre | mC | 6.23(2)  15.09(4)  5.647(19)  90.0  114.2(4)  90.0 | Gypsum |  |
| Metal tolerant (+ Pb) | B_Pb_22_1_7-3 | pre | mC | 6.04(17)  15.2(2)  5.8(3)  88(2)  111(4)  92(2) | Gypsum |  |
| Metal tolerant (+ Pb) | B_Pb_23_1-1 | pre | mC | 6.32(4)  15.05(8)  5.71(3)  90.0(4)  115.5(6)  89.0(5) | Gypsum | 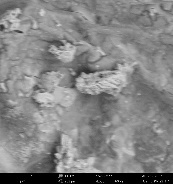 |
| Metal tolerant (+ Pb) | B_Pb_23_1-5 | pre | mC | 6.18(5)  15.36(11)  5.64(3)  90.1(5)  113.6(6)  88.8(6) | Gypsum | 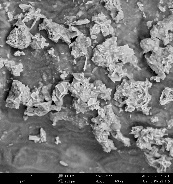 |
| Metal tolerant (+ Pb) | B_Pb_23_2-1 | pre | mC | 6.250(14)  15.210(17)  5.700(17)  89.3(2)  113.9(3)  90.36(15) | Gypsum | 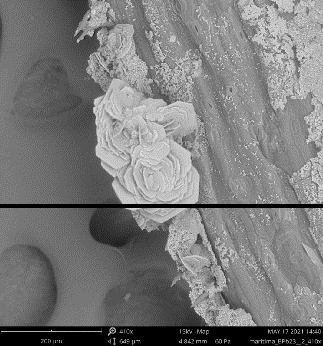 |
| Metal tolerant (+ Pb) | B_Pb_23_2-3 | pre | mC | 6.26(2)  15.23(2)  5.680(10)  90.0  114.5(3)  90.0 | Gypsum |  |
| Metal tolerant (+ Pb) | B_Pb_23_2-6 | full | mC | 6.2783(15)  15.217(3)  5.6738(13)  90  114.21(3)  90 | Gypsum |  |
| Metal tolerant (+ Pb) | B_Pb_24_1_3-1 | pre | oP | 6.426(10)  5.416(5)  9.192(9)  90.0  90.0  90.0 | Niter | 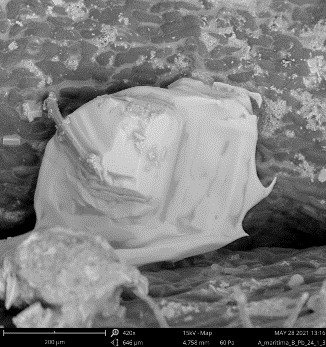 |
| Metal tolerant (+ Pb) | B_Pb_24_1_3-2 | pre | oP | 6.428(18)  5.388(12)  9.162(12)  90.0  90.0  90.0 | Niter |  |
| Metal tolerant (+ Pb) | B_Pb_24_1_3-3 | pre | oP | 6.4351(17)  5.423(3)  9.159(4)  90.0  90.0  90.0 | Niter |  |
| Metal tolerant (+ Pb) | B_Pb_24_1_1-2 | pre | oP | 6.4(4)  5.4(2)  9(1)  90.0  92(7)  90.0 | Niter | 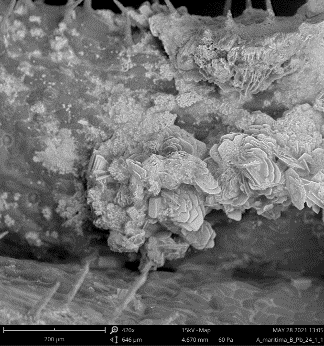 |
| Metal intolerant (+ Pb) | L_Pb_26_2_10-3 | pre | mC | 6.45(11)  14.9(2)  5.9(2)  90.0  114(4)  90.0 | Gypsum | 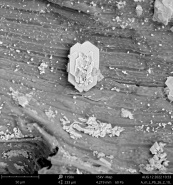 |
| Metal intolerant (+ Pb) | L_Pb_26_2_13-4 | pre | mC | 6.25(4)  15.1(5)  5.66(3)  90(1)  114.4(6)  90(1) | Gypsum | 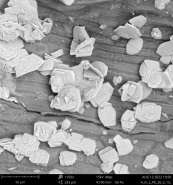 |
| Metal intolerant (+ Pb) | L_Pb_26_2-12 | pre | mC | 6.20(9)  15.18(4)  5.67(3)  89.8(3)  114.3(9)  90.0(6) | Gypsum | 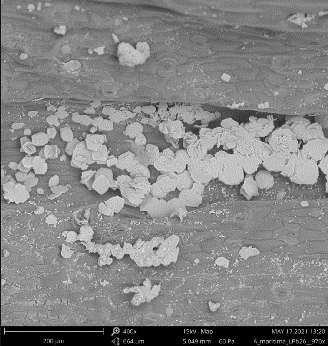 |
| Metal intolerant (+ Pb) | L_Pb_26_2-15 | pre | mC | 6.28(4)  15.14(10)  5.69(3)  91.0(5)  114.8(6)  89.1(5) | Gypsum |  |
| Metal intolerant (+ Pb) | L_Pb_27_1_3-2 | pre | mC | 6.282(7)  15.26(2)  5.697(9)  90.0  114.07(17)  90.0 | Gypsum | 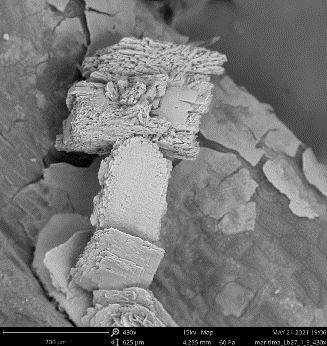 |
| Metal intolerant (+ Pb) | L_Pb_27_1_3-3 | pre | mC | 6.277(9)  15.270(16)  5.664(9)  90.0  114.09(17)  90.0 | Gypsum |  |
| Metal intolerant (+ Pb) | L_Pb_27_1_3-4 | pre | mC | 6.25(3)  15.27(13)  5.65(2)  90.1(5)  114.1(5)  89.6(5) | Gypsum |  |
| Metal intolerant (+ Pb) | L_Pb_27_1_2-1 | pre | oP | 6.439(11)  5.439(16)  9.19(2)  90.0  90.0  90.0 | Niter | 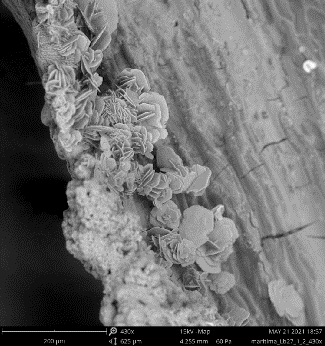 |
| Metal intolerant (+ Pb) | L_Pb_27_1_2-2 | pre | mC | 6.43(6)  15.08(2)  5.62(5)  90.0  113(1)  90.0 | Gypsum |  |
| Metal intolerant (+ Pb) | L_Pb_27_1_2-3 | pre | mC | 6.28(2)  15.19(3)  5.674(13)  90.0  113.9(4)  90.0 | Gypsum |  |
| Metal intolerant (+ Pb) | L_Pb_27_1_2-5 | pre | oP | 6.438(11)  5.409(12)  9.21(3)  90.0  90.0  90.0 | Niter |  |
| Metal intolerant (+ Pb) | L_Pb_27_1_1-1 | pre | oP | 6.275(16)  5.362(11)  9.206(14)  90.0  90.0  90.0 | Niter | 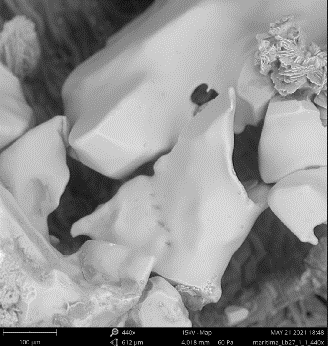 |
| Metal intolerant (+ Pb) | L_Pb_27_1_1-2 | pre | oP | 6.388(10)  5.446(5)  9.216(17)  90.0  90.0  90.0 | Niter |  |
| Metal intolerant (+ Pb) | L_Pb_27_1_1-3 | pre | oP | 6.431(7)  5.404(5)  9.207(10)  90.0  90.0  90.0 | Niter |  |
| Metal intolerant (+ Pb) | L_Pb_27_1_1-4 | pre | oP | 6.432(11)  5.403(12)  9.219(19)  90.0  90.4(2)  90.0 | Niter |  |
| Metal intolerant (+ Pb) | L_Pb_28_1_5-3 | pre | mC | 6.3(3)  15.1(4)  5.66(16)  90(2)  114(4)  90(3) | Gypsum | 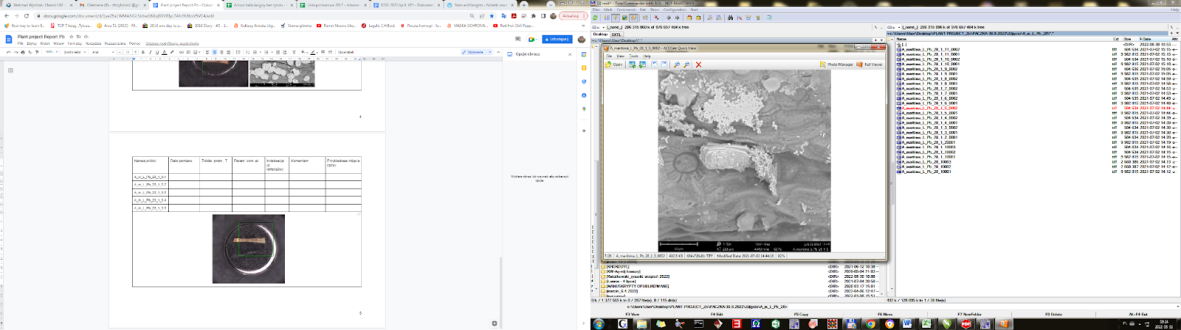 |
| Metal intolerant (+ Pb) | L_Pb_28_1_9-1 | full | mC | 6.2562(14)  15.1356(15)  5.6698(10)  90  114.43(3)  90 | Gypsum | 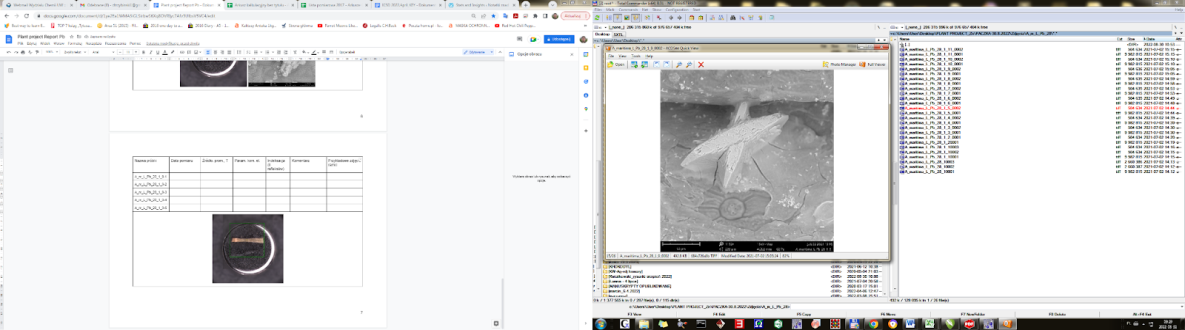 |
| Metal intolerant (+ Pb) | L_Pb_28_1_9-2 | pre | mC | 6.25(4)  15.1(5)  5.66(3)  90(1)  114.4(6)  90(1) | Gypsum |  |
| Metal intolerant (+ Pb) | L_Pb_28_1_9-3 | pre | mC | 6.26(6)  15.10(3)  5.66(4)  90.0  114.5(8)  90.0, | Gypsum |  |
| Metal intolerant (+ Pb) | L_Pb_29_1_8-1 | pre | mC | 6.272(12)  15.14(3)  5.672(10)  90.0  114.8(3)  90.0 | Gypsum | 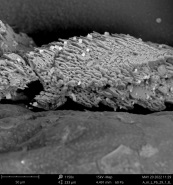 |
| Metal intolerant (+ Pb) | L_Pb_29_1_9-1 | pre | mC | 6.20(13)  15.14(10)  5.75(19)  90.0  115(3)  90.0 | Gypsum | 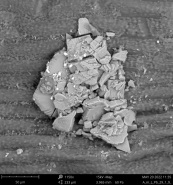 |
| Metal intolerant (+ Pb) | L_Pb_29_1_9-2 | pre | mC | 6.27(6)  15.10(5)  5.67(3)  90.0  114.6(10)  90.0 | Gypsum |  |
| Metal intolerant (+ Pb) | L_Pb_29_1_9-3 | pre | mC | 6.22(6)  15.1(2)  5.68(6)  90.0  114(1)  90.0 | Gypsum |  |
| Metal intolerant (+ Pb) | L_Pb_30_1_1-1 | pre | mC | 6.24(7)  15.28(5)  5.70(5)  90.0  115(1)  90.0 | Gypsum | 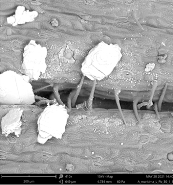 |
| Metal intolerant (+ Pb) | L_Pb_30_1_1-3 | pre | mC | 6.19(5)  15.16(7)  5.85(5)  90.2(5)  113.7(8)  89.3(5) | Gypsum |  |
| Metal intolerant (+ Pb) | L_Pb_30_1_5-2 | pre | mC | 6.291(5)  15.226(14)  5.656(4)  90.0  114.17(8)  90.0 | Gypsum | 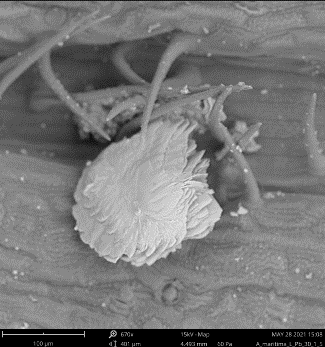 |
| Metal intolerant (+ Pb) | L_Pb_30_1_5-3 | pre | mC | 6.329(19)  15.21(4)  5.700(13)  90.0  114.5(3)  90.0 | Gypsum |  |
| Metal intolerant (+ Pb) | L_Pb_30_1-2 | pre | mC | 6.286(7)  15.305(11)  5.690(6)  90.0  114.23(13)  90.0 | Gypsum | 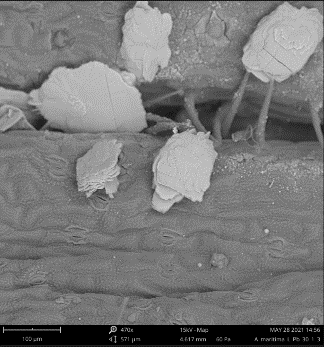 |
| Metal intolerant (+ Pb) | L_Pb_30_1-3 | pre | mC | 6.310(7)  15.245(10)  5.692(7)  90.0  114.28(15)  90.0 | Gypsum |  |
| Metal intolerant (+ Pb) | L_Pb_30_1_2-2 | pre | mC | 6.28(3)  15.24(3)  5.71(3)  90.0  114.5(6)  90.0 | Gypsum | 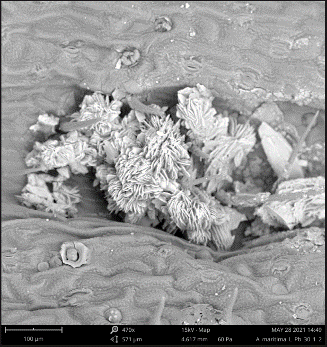 |
| Metal intolerant (+ Pb) | L_Pb_31_1_12-3 | pre | oP | 6.28(4)  5.40(5)  9.14(5)  90.0  90.0  90.0 | Niter | 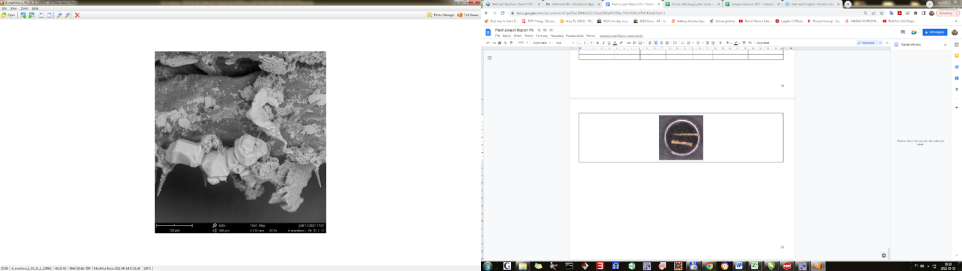 |
| Metal intolerant (+ Pb) | L_Pb_31_2_13-2 | pre | oP | 6.24(3)  5.352(15)  9.27(2)  90.0  90.0  90.0 | Niter | 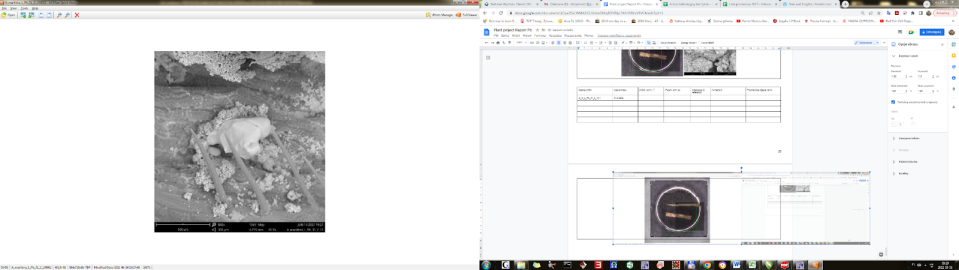 |
| Metal intolerant (+ Pb) | L_Pb_31_2_14-1 | pre | mC | 6.286(9)  15.207(17)  5.700(6)  90.02(11)  114.44(16)  90.02(9) | Gypsum | 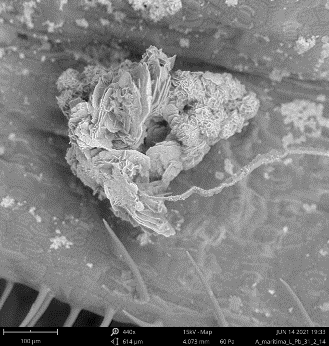 |
| Metal intolerant (+ Pb) | L_Pb_31_2_14-2 | pre | mC | 6.277(15)  15.181(16)  5.687(8)  90.0  114.4(2)  90.0 | Gypsum |  |
| Metal intolerant (+ Pb) | L_Pb_31_2_10-3 | pre | mC | 6.265(7)  15.13(3)  5.659(5)  90.0  114.35(13)  90.0 | Gypsum | 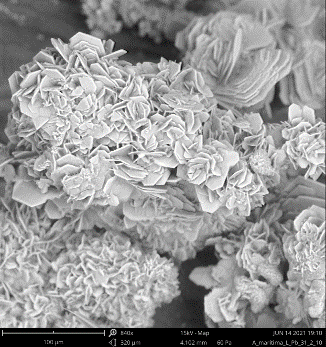 |
| Metal intolerant (+ Pb) | L_Pb_31_1_9-1 | pre | oP | 6.281(3)  5.400(4)  9.126(8)  90.0  90.0  90.0 | Niter | 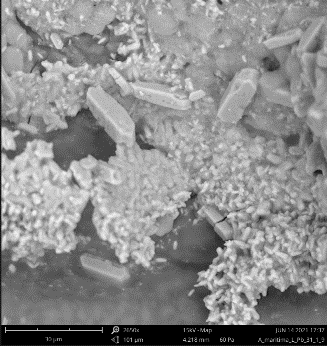 |
| Metal intolerant (+ Pb) | L_Pb_31_1_12-1 | pre | oP | 6.26(2)  5.375(7)  9.192(12)  90.0  90.0  90.0 | Niter | 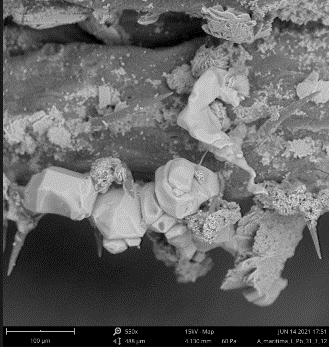 |
| Metal intolerant (+ Pb) | L_Pb_31_1_12-2 | pre | oP | 6.268(3)  5.393(5)  9.142(3)  90.0  90.0  90.0 | Niter |  |
| Metal intolerant (+ Pb) | L_Pb_31_1_12-3 | pre | oP | 6.266(7)  5.370(7)  9.171(9)  90.0  90.0  90.0 | Niter |  |
| Metal intolerant (+ Pb) | L_Pb_31_1_13-1 | full | oP | 6.2730(4)  5.3930(3)  9.1408(7)  90.0  90.0  90.0 | Niter | 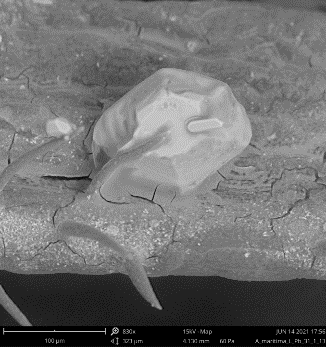 |
| Metal intolerant (+ Pb) | L_Pb_31_2_4-1 | pre | oP | 6.278(14)  5.38(2)  9.191(16)  90.0  90.0  90.0 | Niter | 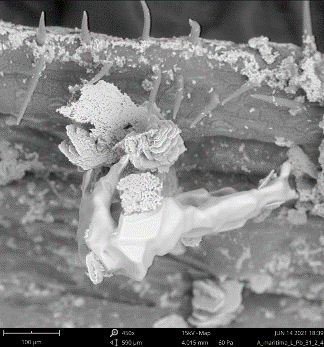 |
| Metal intolerant (+ Pb) | L_Pb_31_2_4-2 | pre | oP | 6.271(11)  5.327(8)  9.285(19)  90.0  90.0  90.0 | Niter |  |

Where: pre – X-Ray diffraction screening; full – complete X-Ray diffraction measurement.

**Table S2.** Bond lengths for gypsum [Å].

| **Atom** | **Atom** | **Length/Å** |  | **Atom** | **Atom** | **Length/Å** |
| --- | --- | --- | --- | --- | --- | --- |
| Ca1 | Ca1^1^ | 4.0415(14) |  | Ca1 | O2^2^ | 2.542(2) |
| Ca1 | Ca1^2^ | 4.0415(14) |  | Ca1 | O2^5^ | 2.542(2) |
| Ca1 | S1^3^ | 3.1283(7) |  | S1 | Ca1^2^ | 3.1283(7) |
| Ca1 | S1^2^ | 3.1283(7) |  | S1 | Ca1^3^ | 3.1283(7) |
| Ca1 | O1^2^ | 2.531(2) |  | S1 | O1^6^ | 1.475(2) |
| Ca1 | O1^4^ | 2.360(2) |  | S1 | O1 | 1.475(2) |
| Ca1 | O1^5^ | 2.531(2) |  | S1 | O2^6^ | 1.478(2) |
| Ca1 | O1 | 2.360(2) |  | S1 | O2 | 1.478(2) |
| Ca1 | O1W | 2.362(2) |  | O1 | Ca1^2^ | 2.531(2) |
| Ca1 | O1W^4^ | 2.362(2) |  | O2 | Ca1^2^ | 2.542(2) |

^1^3/2-X,1/2-Y,2-Z; ^2^1/2-X,1/2-Y,1-Z; ^3^3/2-X,1/2-Y,1-Z; ^4^1-X,+Y,3/2-Z; ^5^1/2+X,1/2-Y,1/2+Z; ^6^1-X,+Y,1/2-Z

**Table S3.** Values of valence angles for gypsum [ º].

| **Atom** | **Atom** | **Atom** | **Angle/˚** |  | **Atom** | **Atom** | **Atom** | **Angle/˚** |
| --- | --- | --- | --- | --- | --- | --- | --- | --- |
| Ca1^1^ | Ca1 | Ca1^2^ | 106.51(4) |  | O1W | Ca1 | Ca1^2^ | 79.67(6) |
| S1^3^ | Ca1 | Ca1^2^ | 60.602(19) |  | O1W^4^ | Ca1 | S1^3^ | 104.04(6) |
| S1^3^ | Ca1 | Ca1^1^ | 118.49(2) |  | O1W | Ca1 | S1^1^ | 104.04(6) |
| S1^1^ | Ca1 | Ca1^2^ | 118.49(2) |  | O1W^4^ | Ca1 | S1^1^ | 76.86(6) |
| S1^1^ | Ca1 | Ca1^1^ | 60.602(19) |  | O1W | Ca1 | S1^3^ | 76.86(6) |
| S1^1^ | Ca1 | S1^3^ | 178.67(4) |  | O1W | Ca1 | O1^5^ | 77.74(8) |
| O1 | Ca1 | Ca1^1^ | 35.66(5) |  | O1W^4^ | Ca1 | O1^5^ | 131.69(8) |
| O1^1^ | Ca1 | Ca1^1^ | 32.94(5) |  | O1W | Ca1 | O1^1^ | 131.69(8) |
| O1^4^ | Ca1 | Ca1^1^ | 91.70(6) |  | O1W^4^ | Ca1 | O1^1^ | 77.74(8) |
| O1 | Ca1 | Ca1^2^ | 91.70(6) |  | O1W^4^ | Ca1 | O1W | 98.01(13) |
| O1^5^ | Ca1 | Ca1^1^ | 115.23(6) |  | O1W^4^ | Ca1 | O2^1^ | 80.01(8) |
| O1^1^ | Ca1 | Ca1^2^ | 115.23(6) |  | O1W | Ca1 | O2^1^ | 76.20(8) |
| O1^5^ | Ca1 | Ca1^2^ | 32.94(5) |  | O1W | Ca1 | O2^5^ | 80.01(8) |
| O1^4^ | Ca1 | Ca1^2^ | 35.66(5) |  | O1W^4^ | Ca1 | O2^5^ | 76.20(8) |
| O1^1^ | Ca1 | S1^1^ | 27.73(5) |  | O2^1^ | Ca1 | Ca1^2^ | 113.76(5) |
| O1^4^ | Ca1 | S1^1^ | 82.89(5) |  | O2^1^ | Ca1 | Ca1^1^ | 88.45(5) |
| O1^1^ | Ca1 | S1^3^ | 151.30(5) |  | O2^5^ | Ca1 | Ca1^1^ | 113.76(5) |
| O1^5^ | Ca1 | S1^3^ | 27.73(5) |  | O2^5^ | Ca1 | Ca1^2^ | 88.45(5) |
| O1^4^ | Ca1 | S1^3^ | 96.23(5) |  | O2^1^ | Ca1 | S1^3^ | 153.06(5) |
| O1 | Ca1 | S1^1^ | 96.23(5) |  | O2^5^ | Ca1 | S1^3^ | 27.85(5) |
| O1^5^ | Ca1 | S1^1^ | 151.30(5) |  | O2^5^ | Ca1 | S1^1^ | 153.06(5) |
| O1 | Ca1 | S1^3^ | 82.89(5) |  | O2^1^ | Ca1 | S1^1^ | 27.85(5) |
| O1^1^ | Ca1 | O1^5^ | 139.61(10) |  | O2^5^ | Ca1 | O2^1^ | 143.39(11) |
| O1 | Ca1 | O1^4^ | 98.26(11) |  | Ca1^3^ | S1 | Ca1^1^ | 178.67(4) |
| O1 | Ca1 | O1^1^ | 68.60(8) |  | O1 | S1 | Ca1^1^ | 52.97(8) |
| O1^4^ | Ca1 | O1^5^ | 68.60(8) |  | O1^6^ | S1 | Ca1^3^ | 52.97(8) |
| O1 | Ca1 | O1^5^ | 85.02(5) |  | O1^6^ | S1 | Ca1^1^ | 127.99(9) |
| O1^4^ | Ca1 | O1^1^ | 85.02(5) |  | O1 | S1 | Ca1^3^ | 127.99(9) |
| O1^4^ | Ca1 | O1W | 85.44(8) |  | O1^6^ | S1 | O1 | 110.84(17) |
| O1 | Ca1 | O1W | 159.71(7) |  | O1 | S1 | O2^6^ | 111.03(11) |
| O1^4^ | Ca1 | O1W^4^ | 159.71(7) |  | O1 | S1 | O2 | 106.38(11) |
| O1 | Ca1 | O1W^4^ | 85.44(8) |  | O1^6^ | S1 | O2^6^ | 106.38(11) |
| O1^5^ | Ca1 | O2^1^ | 141.46(6) |  | O1^6^ | S1 | O2 | 111.03(11) |
| O1 | Ca1 | O2^5^ | 81.44(7) |  | O2 | S1 | Ca1^3^ | 125.63(8) |
| O1 | Ca1 | O2^1^ | 124.04(7) |  | O2^6^ | S1 | Ca1^1^ | 125.63(8) |
| O1^5^ | Ca1 | O2^5^ | 55.57(6) |  | O2 | S1 | Ca1^1^ | 53.44(8) |
| O1^4^ | Ca1 | O2^5^ | 124.04(7) |  | O2^6^ | S1 | Ca1^3^ | 53.44(8) |
| O1^4^ | Ca1 | O2^1^ | 81.44(7) |  | O2 | S1 | O2^6^ | 111.25(18) |
| O1^1^ | Ca1 | O2^5^ | 141.46(6) |  | Ca1 | O1 | Ca1^1^ | 111.40(8) |
| O1^1^ | Ca1 | O2^1^ | 55.57(6) |  | S1 | O1 | Ca1^1^ | 99.30(11) |
| O1W^4^ | Ca1 | Ca1^1^ | 79.67(6) |  | S1 | O1 | Ca1 | 148.89(12) |
| O1W | Ca1 | Ca1^1^ | 164.63(6) |  | S1 | O2 | Ca1^1^ | 98.71(10) |
| O1W^4^ | Ca1 | Ca1^2^ | 164.63(6) |  |  |  |  |  |

^1^1/2-X,1/2-Y,1-Z; ^2^3/2-X,1/2-Y,2-Z; ^3^3/2-X,1/2-Y,1-Z; ^4^1-X,+Y,3/2-Z; ^5^1/2+X,1/2-Y,1/2+Z; ^6^1-X,+Y,1/2-Z

**Table S4.** Values of torsion angles for gypsum [ º].

| **A** | **B** | **C** | **D** | **Angle/˚** |  | **A** | **B** | **C** | **D** | **Angle/˚** |
| --- | --- | --- | --- | --- | --- | --- | --- | --- | --- | --- |
| Ca1^1^ | S1 | O1 | Ca1 | 170.8(3) |  | O1^3^ | S1 | O2 | Ca1^1^ | -122.40(11) |
| Ca1^2^ | S1 | O1 | Ca1 | -8.0(3) |  | O2 | S1 | O1 | Ca1 | 172.5(2) |
| Ca1^2^ | S1 | O1 | Ca1^1^ | -178.83(4) |  | O2^3^ | S1 | O1 | Ca1^1^ | -119.45(11) |
| Ca1^2^ | S1 | O2 | Ca1^1^ | 178.82(4) |  | O2^3^ | S1 | O1 | Ca1 | 51.3(3) |
| O1^3^ | S1 | O1 | Ca1^1^ | 122.53(11) |  | O2 | S1 | O1 | Ca1^1^ | 1.73(14) |
| O1^3^ | S1 | O1 | Ca1 | -66.7(2) |  | O2^3^ | S1 | O2 | Ca1^1^ | 119.32(10) |
| O1 | S1 | O2 | Ca1^1^ | -1.72(14) |  |  |  |  |  |  |

^1^1/2-X,1/2-Y,1-Z; ^2^3/2-X,1/2-Y,1-Z; ^3^1-X,+Y,1/2-Z

**Table S5.** Bond lengths for niter [Å].

| **Atom** | **Atom** | **Length/Å** |  | **Atom** | **Atom** | **Length/Å** |
| --- | --- | --- | --- | --- | --- | --- |
| O2 | K1^1^ | 2.8031(12) |  | K1 | O1^5^ | 2.8464(8) |
| O2 | K1^2^ | 2.8879(5) |  | K1 | O1^4^ | 2.8094(9) |
| O2 | K1^3^ | 2.8879(5) |  | K1 | O1^8^ | 2.8464(8) |
| O2 | N1 | 1.2568(19) |  | K1 | O1^9^ | 2.8094(9) |
| O1 | K1 | 2.8601(8) |  | K1 | O1^10^ | 2.8602(8) |
| O1 | K1^2^ | 2.8465(8) |  | K1 | N1 | 3.2363(14) |
| O1 | K1^4^ | 2.8094(9) |  | K1 | N1^5^ | 3.2471(8) |
| O1 | N1 | 1.2544(11) |  | K1 | N1^7^ | 3.2471(8) |
| K1 | O2^5^ | 2.8879(5) |  | N1 | O1^10^ | 1.2544(11) |
| K1 | O2^6^ | 2.8031(12) |  | N1 | K1^2^ | 3.2471(8) |
| K1 | O2^7^ | 2.8879(5) |  | N1 | K1^3^ | 3.2471(8) |

^1^1/2+X,+Y,1/2-Z; ^2^3/2-X,1-Y,-1/2+Z; ^3^3/2-X,-Y,-1/2+Z; ^4^2-X,1-Y,1-Z; ^5^3/2-X,1-Y,1/2+Z; ^6^-1/2+X,+Y,1/2-Z; ^7^3/2-X,-Y,1/2+Z; ^8^3/2-X,-1/2+Y,1/2+Z; ^9^2-X,-1/2+Y,1-Z; ^10^+X,1/2-Y,+Z

**Table S6.** Values of valence angles for niter [ º].

| **Atom** | **Atom** | **Atom** | **Angle/˚** |  | **Atom** | **Atom** | **Atom** | **Angle/˚** |
| --- | --- | --- | --- | --- | --- | --- | --- | --- |
| K1^1^ | O2 | K1^2^ | 102.92(2) |  | O1^7^ | K1 | O1^9^ | 167.28(3) |
| K1^1^ | O2 | K1^3^ | 102.92(2) |  | O1^4^ | K1 | O1 | 79.32(3) |
| K1^2^ | O2 | K1^3^ | 138.05(5) |  | O1^8^ | K1 | O1^10^ | 72.592(18) |
| N1 | O2 | K1^1^ | 127.45(9) |  | O1^7^ | K1 | O1 | 123.067(8) |
| N1 | O2 | K1^3^ | 94.93(4) |  | O1^8^ | K1 | O1 | 104.491(18) |
| N1 | O2 | K1^2^ | 94.93(4) |  | O1^7^ | K1 | O1^10^ | 68.85(3) |
| K1^4^ | O1 | K1 | 100.68(3) |  | O1^10^ | K1 | O1 | 167.28(3) |
| K1^4^ | O1 | K1^3^ | 101.22(3) |  | O1^4^ | K1 | O1^7^ | 72.592(18) |
| K1^3^ | O1 | K1 | 136.82(3) |  | O1^4^ | K1 | O1^8^ | 69.89(4) |
| N1 | O1 | K1^3^ | 96.98(7) |  | O1 | K1 | O1^9^ | 44.69(3) |
| N1 | O1 | K1^4^ | 131.36(8) |  | O1^7^ | K1 | N1 | 144.654(18) |
| N1 | O1 | K1 | 95.76(7) |  | O1^9^ | K1 | N1 | 22.684(17) |
| O2^5^ | K1 | O2^6^ | 77.08(2) |  | O1^9^ | K1 | N1^7^ | 144.79(3) |
| O2^6^ | K1 | O2^7^ | 138.05(5) |  | O1 | K1 | N1 | 22.684(17) |
| O2^5^ | K1 | O2^7^ | 77.08(2) |  | O1 | K1 | N1^7^ | 100.70(3) |
| O2^5^ | K1 | O1^8^ | 142.462(19) |  | O1 | K1 | N1^6^ | 144.79(3) |
| O2^5^ | K1 | O1^9^ | 74.19(3) |  | O1^10^ | K1 | N1 | 144.654(18) |
| O2^5^ | K1 | O1^4^ | 142.462(19) |  | O1^8^ | K1 | N1 | 95.30(3) |
| O2^5^ | K1 | O1 | 74.19(3) |  | O1^4^ | K1 | N1^6^ | 124.21(3) |
| O2^5^ | K1 | O1^10^ | 100.36(3) |  | O1^7^ | K1 | N1^6^ | 90.88(3) |
| O2^5^ | K1 | O1^7^ | 100.36(3) |  | O1^8^ | K1 | N1^7^ | 124.21(3) |
| O2^7^ | K1 | N1^6^ | 128.92(3) |  | O1^10^ | K1 | N1^6^ | 22.55(3) |
| O2^7^ | K1 | N1^7^ | 22.68(3) |  | O1^9^ | K1 | N1^6^ | 100.70(3) |
| O2^6^ | K1 | N1 | 100.37(2) |  | O1^4^ | K1 | N1 | 95.30(3) |
| O2^7^ | K1 | N1 | 100.37(2) |  | O1^7^ | K1 | N1^7^ | 22.55(3) |
| O2^5^ | K1 | N1^7^ | 92.09(3) |  | O1^4^ | K1 | N1^7^ | 67.10(3) |
| O2^6^ | K1 | N1^7^ | 128.92(3) |  | O1^10^ | K1 | N1^7^ | 90.88(3) |
| O2^6^ | K1 | N1^6^ | 22.68(3) |  | O1^8^ | K1 | N1^6^ | 67.10(3) |
| O2^5^ | K1 | N1 | 68.85(4) |  | N1 | K1 | N1^6^ | 122.12(2) |
| O2^5^ | K1 | N1^6^ | 92.09(3) |  | N1^7^ | K1 | N1^6^ | 112.29(4) |
| O1 | K1 | O2^7^ | 80.28(3) |  | N1 | K1 | N1^7^ | 122.12(2) |
| O1^4^ | K1 | O2^6^ | 140.37(3) |  | O2 | N1 | K1 | 161.26(10) |
| O1^9^ | K1 | O2^6^ | 80.28(3) |  | O2 | N1 | K1^2^ | 62.39(3) |
| O1^10^ | K1 | O2^6^ | 44.54(3) |  | O2 | N1 | K1^3^ | 62.39(3) |
| O1 | K1 | O2^6^ | 122.76(3) |  | O1^9^ | N1 | O2 | 119.90(7) |
| O1^7^ | K1 | O2^7^ | 44.54(3) |  | O1 | N1 | O2 | 119.90(7) |
| O1^9^ | K1 | O2^7^ | 122.76(3) |  | O1 | N1 | O1^9^ | 120.18(13) |
| O1^10^ | K1 | O2^7^ | 110.06(3) |  | O1 | N1 | K1^2^ | 161.24(9) |
| O1^4^ | K1 | O2^7^ | 72.57(3) |  | O1 | N1 | K1 | 61.56(7) |
| O1^8^ | K1 | O2^6^ | 72.57(3) |  | O1^9^ | N1 | K1 | 61.56(7) |
| O1^8^ | K1 | O2^7^ | 140.37(3) |  | O1 | N1 | K1^3^ | 60.47(5) |
| O1^7^ | K1 | O2^6^ | 110.06(3) |  | O1^9^ | N1 | K1^2^ | 60.47(5) |
| O1^8^ | K1 | O1^7^ | 110.39(2) |  | O1^9^ | N1 | K1^3^ | 161.24(9) |
| O1^8^ | K1 | O1^9^ | 79.32(3) |  | K1^2^ | N1 | K1^3^ | 112.29(4) |
| O1^4^ | K1 | O1^9^ | 104.491(18) |  | K1 | N1 | K1^2^ | 109.86(3) |
| O1^10^ | K1 | O1^9^ | 123.067(8) |  | K1 | N1 | K1^3^ | 109.86(3) |
| O1^4^ | K1 | O1^10^ | 110.39(2) |  |  |  |  |  |

^1^1/2+X,+Y,1/2-Z; ^2^3/2-X,-Y,-1/2+Z; ^3^3/2-X,1-Y,-1/2+Z; ^4^2-X,1-Y,1-Z; ^5^-1/2+X,+Y,1/2-Z; ^6^3/2-X,-Y,1/2+Z; ^7^3/2-X,1-Y,1/2+Z; ^8^2-X,-1/2+Y,1-Z; ^9^+X,1/2-Y,+Z; ^10^3/2-X,-1/2+Y,1/2+Z

**Table S7.** Values of torsion angles for niter [ º].

| **A** | **B** | **C** | **D** | **Angle/˚** |  | **A** | **B** | **C** | **D** | **Angle/˚** |
| --- | --- | --- | --- | --- | --- | --- | --- | --- | --- | --- |
| K1^1^ | O2 | N1 | O1 | 90.86(12) |  | K1 | O1 | N1 | O2 | 158.58(12) |
| K1^1^ | O2 | N1 | O1^2^ | -90.86(12) |  | K1^3^ | O1 | N1 | O2 | 19.92(13) |
| K1^3^ | O2 | N1 | O1^2^ | 158.72(11) |  | K1^5^ | O1 | N1 | O2 | -91.73(14) |
| K1^3^ | O2 | N1 | O1 | -19.55(13) |  | K1 | O1 | N1 | O1^2^ | -19.69(13) |
| K1^4^ | O2 | N1 | O1 | -158.73(11) |  | K1^3^ | O1 | N1 | O1^2^ | -158.35(11) |
| K1^4^ | O2 | N1 | O1^2^ | 19.55(13) |  | K1^5^ | O1 | N1 | O1^2^ | 89.99(14) |
| K1^1^ | O2 | N1 | K1^3^ | 110.41(3) |  | K1^3^ | O1 | N1 | K1 | -138.65(4) |
| K1^4^ | O2 | N1 | K1 | -69.59(3) |  | K1^5^ | O1 | N1 | K1 | 109.69(8) |
| K1^1^ | O2 | N1 | K1^4^ | -110.41(3) |  | K1^5^ | O1 | N1 | K1^3^ | -111.66(8) |
| K1^3^ | O2 | N1 | K1^4^ | 139.17(6) |  | K1^3^ | O1 | N1 | K1^4^ | -71.8(3) |
| K1^3^ | O2 | N1 | K1 | 69.59(3) |  | K1 | O1 | N1 | K1^4^ | 66.8(3) |
| K1^1^ | O2 | N1 | K1 | 180.0 |  | K1^5^ | O1 | N1 | K1^4^ | 176.53(18) |
| K1^4^ | O2 | N1 | K1^3^ | -139.17(6) |  | K1 | O1 | N1 | K1^3^ | 138.65(4) |

^1^1/2+X,+Y,1/2-Z; ^2^+X,1/2-Y,+Z; ^3^3/2-X,1-Y,-1/2+Z; ^4^3/2-X,-Y,-1/2+Z; ^5^2-X,1-Y,1-Z

**Table S8.** Bond lengths for syngenite [Å].

| **Atom** | **Atom** | **Length/Å** |  | **Atom** | **Atom** | **Length/Å** |
| --- | --- | --- | --- | --- | --- | --- |
| Ca1 | O1^1^ | 2.5457(19) |  | O2 | K1^11^ | 2.858(2) |
| Ca1 | O1 | 2.5458(19) |  | O2 | S1 | 1.480(3) |
| Ca1 | O1W | 2.461(3) |  | O3 | K1^12^ | 2.850(2) |
| Ca1 | O4 | 2.5185(18) |  | O3 | K1^11^ | 2.955(2) |
| Ca1 | O4^2^ | 2.4427(18) |  | O3 | K1^10^ | 2.955(2) |
| Ca1 | O4^3^ | 2.4427(18) |  | O3 | K1^8^ | 2.850(2) |
| Ca1 | O4^1^ | 2.5185(18) |  | O3 | S1 | 1.480(3) |
| Ca1 | O5^4^ | 2.401(3) |  | O4 | Ca1^2^ | 2.4427(18) |
| Ca1 | O6^4^ | 2.703(3) |  | O4 | K1^12^ | 3.3844(19) |
| Ca1 | S1 | 3.1441(11) |  | O4 | S2 | 1.4780(18) |
| Ca1 | S2 | 3.1105(10) |  | O5 | Ca1^13^ | 2.401(3) |
| Ca1 | S2^4^ | 3.1267(10) |  | O5 | K1^8^ | 2.671(2) |
| K1 | K1^1^ | 3.4591(11) |  | O5 | K1^12^ | 2.671(2) |
| K1 | O1^5^ | 2.9452(19) |  | O5 | S2 | 1.479(3) |
| K1 | O1W | 2.962(2) |  | O6 | Ca1^13^ | 2.703(3) |
| K1 | O2^6^ | 2.900(2) |  | O6 | K1^13^ | 2.745(2) |
| K1 | O2^7^ | 2.858(2) |  | O6 | K1^14^ | 2.745(2) |
| K1 | O3^7^ | 2.955(2) |  | O6 | S2 | 1.470(3) |
| K1 | O3^8^ | 2.850(2) |  | S1 | K1^11^ | 3.3371(9) |
| K1 | O4^3^ | 3.3844(19) |  | S1 | K1^12^ | 3.7717(9) |
| K1 | O5^8^ | 2.671(2) |  | S1 | K1^6^ | 3.4703(9) |
| K1 | O6^4^ | 2.745(2) |  | S1 | K1^10^ | 3.3371(9) |
| K1 | S1^7^ | 3.3371(9) |  | S1 | K1^9^ | 3.4703(9) |
| K1 | S1^6^ | 3.4703(9) |  | S1 | K1^8^ | 3.7717(10) |
| O1 | K1^9^ | 2.9452(19) |  | S1 | O1^1^ | 1.4814(18) |
| O1 | S1 | 1.4814(18) |  | S2 | Ca1^13^ | 3.1267(10) |
| O1W | K1^1^ | 2.962(2) |  | S2 | K1^8^ | 3.6156(9) |
| O2 | K1^10^ | 2.858(2) |  | S2 | K1^12^ | 3.6156(9) |
| O2 | K1^6^ | 2.900(2) |  | S2 | O4^1^ | 1.4780(18) |
| O2 | K1^9^ | 2.900(2) |  |  |  |  |

^1^+X,1/2-Y,+Z; ^2^1-X,1-Y,1-Z; ^3^1-X,-1/2+Y,1-Z; ^4^1+X,+Y,+Z; ^5^2-X,-1/2+Y,1-Z; ^6^2-X,-Y,1-Z; ^7^+X,+Y,-1+Z; ^8^1-X,-Y,1-Z; ^9^2-X,1/2+Y,1-Z; ^10^+X,+Y,1+Z; ^11^+X,1/2-Y,1+Z; ^12^1-X,1/2+Y,1-Z; ^13^-1+X,+Y,+Z; ^14^-1+X,1/2-Y,+Z

**Table S9.** Values of valence angles for syngenite [ º].

| **Atom** | **Atom** | **Atom** | **Angle/˚** |  | **Atom** | **Atom** | **Atom** | **Angle/˚** |
| --- | --- | --- | --- | --- | --- | --- | --- | --- |
| O1^1^ | Ca1 | O1 | 55.41(8) |  | S1^7^ | K1 | O4^4^ | 152.57(4) |
| O1 | Ca1 | O6^2^ | 122.82(6) |  | S1^7^ | K1 | S1^5^ | 98.11(2) |
| O1^1^ | Ca1 | O6^2^ | 122.82(6) |  | Ca1 | O1 | K1^9^ | 101.56(6) |
| O1 | Ca1 | S1 | 27.71(4) |  | S1 | O1 | Ca1 | 99.23(9) |
| O1^1^ | Ca1 | S1 | 27.71(4) |  | S1 | O1 | K1^9^ | 97.74(9) |
| O1^1^ | Ca1 | S2 | 87.83(5) |  | Ca1 | O1W | K1^1^ | 102.62(8) |
| O1^1^ | Ca1 | S2^2^ | 98.61(5) |  | Ca1 | O1W | K1 | 102.62(8) |
| O1 | Ca1 | S2 | 87.83(5) |  | K1^1^ | O1W | K1 | 71.46(6) |
| O1 | Ca1 | S2^2^ | 98.61(5) |  | K1^9^ | O2 | K1^5^ | 78.31(7) |
| O1W | Ca1 | O1^1^ | 148.16(5) |  | K1^10^ | O2 | K1^5^ | 101.60(2) |
| O1W | Ca1 | O1 | 148.16(5) |  | K1^11^ | O2 | K1^9^ | 101.60(2) |
| O1W | Ca1 | O4^1^ | 78.15(7) |  | K1^11^ | O2 | K1^5^ | 164.88(10) |
| O1W | Ca1 | O4 | 78.15(7) |  | K1^11^ | O2 | K1^10^ | 74.48(7) |
| O1W | Ca1 | O6^2^ | 68.61(8) |  | K1^10^ | O2 | K1^9^ | 164.88(10) |
| O1W | Ca1 | S1 | 162.93(7) |  | S1 | O2 | K1^9^ | 99.66(11) |
| O1W | Ca1 | S2 | 76.09(6) |  | S1 | O2 | K1^11^ | 95.27(11) |
| O1W | Ca1 | S2^2^ | 96.63(6) |  | S1 | O2 | K1^5^ | 99.66(11) |
| O4^3^ | Ca1 | O1^1^ | 129.48(6) |  | S1 | O2 | K1^10^ | 95.27(11) |
| O4^1^ | Ca1 | O1^1^ | 75.05(6) |  | K1^8^ | O3 | K1^10^ | 96.08(3) |
| O4 | Ca1 | O1 | 75.05(6) |  | K1^11^ | O3 | K1^10^ | 71.66(7) |
| O4^1^ | Ca1 | O1 | 100.28(6) |  | K1^8^ | O3 | K1^12^ | 79.98(7) |
| O4^4^ | Ca1 | O1 | 129.48(6) |  | K1^8^ | O3 | K1^11^ | 149.14(10) |
| O4 | Ca1 | O1^1^ | 100.28(6) |  | K1^12^ | O3 | K1^10^ | 149.14(10) |
| O4^3^ | Ca1 | O1 | 74.35(6) |  | K1^12^ | O3 | K1^11^ | 96.08(3) |
| O4^4^ | Ca1 | O1^1^ | 74.35(6) |  | S1 | O3 | K1^12^ | 117.68(11) |
| O4^3^ | Ca1 | O1W | 80.12(5) |  | S1 | O3 | K1^10^ | 91.43(11) |
| O4^4^ | Ca1 | O1W | 80.12(5) |  | S1 | O3 | K1^11^ | 91.43(11) |
| O4^3^ | Ca1 | O4 | 68.65(7) |  | S1 | O3 | K1^8^ | 117.68(11) |
| O4^4^ | Ca1 | O4^1^ | 68.65(7) |  | Ca1^3^ | O4 | Ca1 | 111.35(7) |
| O4^1^ | Ca1 | O4 | 56.00(8) |  | Ca1^3^ | O4 | K1^12^ | 92.04(5) |
| O4^4^ | Ca1 | O4^3^ | 153.70(9) |  | Ca1 | O4 | K1^12^ | 114.95(6) |
| O4^4^ | Ca1 | O4 | 123.38(5) |  | S2 | O4 | Ca1^3^ | 147.00(11) |
| O4^3^ | Ca1 | O4^1^ | 123.38(5) |  | S2 | O4 | Ca1 | 98.87(9) |
| O4^4^ | Ca1 | O6^2^ | 78.36(4) |  | S2 | O4 | K1^12^ | 86.76(8) |
| O4 | Ca1 | O6^2^ | 136.45(6) |  | Ca1^13^ | O5 | K1^8^ | 114.24(7) |
| O4^1^ | Ca1 | O6^2^ | 136.45(6) |  | Ca1^13^ | O5 | K1^12^ | 114.24(7) |
| O4^3^ | Ca1 | O6^2^ | 78.36(4) |  | K1^12^ | O5 | K1^8^ | 86.59(8) |
| O4^3^ | Ca1 | S1 | 102.00(4) |  | S2 | O5 | Ca1^13^ | 104.90(14) |
| O4 | Ca1 | S1 | 86.80(5) |  | S2 | O5 | K1^12^ | 118.37(9) |
| O4^4^ | Ca1 | S1 | 102.00(4) |  | S2 | O5 | K1^8^ | 118.37(9) |
| O4^1^ | Ca1 | S1 | 86.80(5) |  | Ca1^13^ | O6 | K1^14^ | 102.34(7) |
| O4^3^ | Ca1 | S2 | 95.96(4) |  | Ca1^13^ | O6 | K1^13^ | 102.34(7) |
| O4^1^ | Ca1 | S2^2^ | 151.25(4) |  | K1^13^ | O6 | K1^14^ | 78.11(7) |
| O4^3^ | Ca1 | S2^2^ | 82.61(4) |  | S2 | O6 | Ca1^13^ | 92.24(13) |
| O4 | Ca1 | S2 | 28.00(4) |  | S2 | O6 | K1^14^ | 137.53(6) |
| O4 | Ca1 | S2^2^ | 151.25(4) |  | S2 | O6 | K1^13^ | 137.53(6) |
| O4^4^ | Ca1 | S2^2^ | 82.61(4) |  | Ca1 | S1 | K1^8^ | 92.12(2) |
| O4^4^ | Ca1 | S2 | 95.96(4) |  | Ca1 | S1 | K1^5^ | 80.06(2) |
| O4^1^ | Ca1 | S2 | 28.00(4) |  | Ca1 | S1 | K1^10^ | 148.762(12) |
| O5^2^ | Ca1 | O1^1^ | 74.59(7) |  | Ca1 | S1 | K1^11^ | 148.762(12) |
| O5^2^ | Ca1 | O1 | 74.59(7) |  | Ca1 | S1 | K1^12^ | 92.12(2) |
| O5^2^ | Ca1 | O1W | 123.83(9) |  | Ca1 | S1 | K1^9^ | 80.06(2) |
| O5^2^ | Ca1 | O4^4^ | 88.36(5) |  | K1^11^ | S1 | K1^10^ | 62.43(2) |
| O5^2^ | Ca1 | O4 | 145.86(6) |  | K1^9^ | S1 | K1^8^ | 171.51(3) |
| O5^2^ | Ca1 | O4^1^ | 145.86(6) |  | K1^5^ | S1 | K1^12^ | 171.51(3) |
| O5^2^ | Ca1 | O4^3^ | 88.36(5) |  | K1^11^ | S1 | K1^5^ | 113.95(3) |
| O5^2^ | Ca1 | O6^2^ | 55.22(8) |  | K1^11^ | S1 | K1^9^ | 81.89(2) |
| O5^2^ | Ca1 | S1 | 73.25(6) |  | K1^9^ | S1 | K1^12^ | 118.462(16) |
| O5^2^ | Ca1 | S2^2^ | 27.20(6) |  | K1^8^ | S1 | K1^12^ | 58.10(2) |
| O5^2^ | Ca1 | S2 | 160.08(7) |  | K1^11^ | S1 | K1^8^ | 103.65(2) |
| O6^2^ | Ca1 | S1 | 128.46(6) |  | K1^10^ | S1 | K1^12^ | 103.65(2) |
| O6^2^ | Ca1 | S2 | 144.70(6) |  | K1^10^ | S1 | K1^5^ | 81.89(2) |
| O6^2^ | Ca1 | S2^2^ | 28.02(6) |  | K1^9^ | S1 | K1^5^ | 63.70(2) |
| S2 | Ca1 | S1 | 86.84(3) |  | K1^11^ | S1 | K1^12^ | 74.495(19) |
| S2^2^ | Ca1 | S1 | 100.44(3) |  | K1^10^ | S1 | K1^8^ | 74.495(19) |
| S2 | Ca1 | S2^2^ | 172.72(4) |  | K1^10^ | S1 | K1^9^ | 113.95(3) |
| K1^1^ | K1 | S1^5^ | 121.851(12) |  | K1^5^ | S1 | K1^8^ | 118.462(16) |
| O1^6^ | K1 | K1^1^ | 102.70(4) |  | O1 | S1 | Ca1 | 53.06(8) |
| O1^6^ | K1 | O1W | 138.43(6) |  | O1^1^ | S1 | Ca1 | 53.05(8) |
| O1^6^ | K1 | O3^7^ | 128.20(6) |  | O1 | S1 | K1^9^ | 57.24(8) |
| O1^6^ | K1 | O4^4^ | 100.04(5) |  | O1^1^ | S1 | K1^11^ | 158.07(8) |
| O1^6^ | K1 | S1^5^ | 25.02(4) |  | O1^1^ | S1 | K1^10^ | 95.71(8) |
| O1^6^ | K1 | S1^7^ | 102.49(4) |  | O1 | S1 | K1^5^ | 107.59(8) |
| O1W | K1 | K1^1^ | 54.27(3) |  | O1^1^ | S1 | K1^5^ | 57.24(8) |
| O1W | K1 | O4^4^ | 59.19(5) |  | O1 | S1 | K1^8^ | 115.26(8) |
| O1W | K1 | S1^5^ | 162.19(6) |  | O1^1^ | S1 | K1^8^ | 69.56(8) |
| O1W | K1 | S1^7^ | 93.42(5) |  | O1 | S1 | K1^12^ | 69.56(8) |
| O2^5^ | K1 | K1^1^ | 129.16(3) |  | O1^1^ | S1 | K1^12^ | 115.26(8) |
| O2^7^ | K1 | K1^1^ | 52.76(3) |  | O1^1^ | S1 | K1^9^ | 107.59(8) |
| O2^5^ | K1 | O1^6^ | 49.03(6) |  | O1 | S1 | K1^10^ | 158.07(8) |
| O2^7^ | K1 | O1^6^ | 79.49(6) |  | O1 | S1 | K1^11^ | 95.71(8) |
| O2^5^ | K1 | O1W | 172.51(7) |  | O1 | S1 | O1^1^ | 106.07(15) |
| O2^7^ | K1 | O1W | 102.69(5) |  | O2 | S1 | Ca1 | 126.42(12) |
| O2^7^ | K1 | O2^5^ | 78.40(2) |  | O2 | S1 | K1^10^ | 58.52(9) |
| O2^7^ | K1 | O3^7^ | 49.08(7) |  | O2 | S1 | K1^12^ | 132.91(9) |
| O2^5^ | K1 | O3^7^ | 105.96(7) |  | O2 | S1 | K1^9^ | 55.47(8) |
| O2^7^ | K1 | O4^4^ | 152.04(5) |  | O2 | S1 | K1^11^ | 58.52(9) |
| O2^5^ | K1 | O4^4^ | 122.53(5) |  | O2 | S1 | K1^8^ | 132.91(9) |
| O2^5^ | K1 | S1^5^ | 24.87(5) |  | O2 | S1 | K1^5^ | 55.47(8) |
| O2^7^ | K1 | S1^7^ | 26.21(5) |  | O2 | S1 | O1 | 109.98(10) |
| O2^7^ | K1 | S1^5^ | 82.59(5) |  | O2 | S1 | O1^1^ | 109.98(10) |
| O2^5^ | K1 | S1^7^ | 84.39(5) |  | O3 | S1 | Ca1 | 124.20(12) |
| O3^7^ | K1 | K1^1^ | 54.17(3) |  | O3 | S1 | K1^11^ | 62.26(9) |
| O3^8^ | K1 | K1^1^ | 129.99(4) |  | O3 | S1 | K1^8^ | 41.99(8) |
| O3^8^ | K1 | O1^6^ | 126.00(5) |  | O3 | S1 | K1^9^ | 141.72(6) |
| O3^8^ | K1 | O1W | 89.16(6) |  | O3 | S1 | K1^10^ | 62.26(9) |
| O3^7^ | K1 | O1W | 70.19(7) |  | O3 | S1 | K1^12^ | 41.99(8) |
| O3^8^ | K1 | O2^5^ | 83.98(6) |  | O3 | S1 | K1^5^ | 141.72(6) |
| O3^8^ | K1 | O2^7^ | 120.57(7) |  | O3 | S1 | O1^1^ | 110.70(10) |
| O3^8^ | K1 | O3^7^ | 83.92(3) |  | O3 | S1 | O1 | 110.70(10) |
| O3^7^ | K1 | O4^4^ | 127.56(6) |  | O3 | S1 | O2 | 109.39(16) |
| O3^8^ | K1 | O4^4^ | 82.62(6) |  | Ca1 | S2 | Ca1^13^ | 172.72(4) |
| O3^8^ | K1 | S1^5^ | 102.90(4) |  | Ca1 | S2 | K1^8^ | 95.72(2) |
| O3^7^ | K1 | S1^7^ | 26.31(5) |  | Ca1^13^ | S2 | K1^8^ | 78.03(2) |
| O3^7^ | K1 | S1^5^ | 123.53(5) |  | Ca1 | S2 | K1^12^ | 95.72(2) |
| O3^8^ | K1 | S1^7^ | 96.44(5) |  | Ca1^13^ | S2 | K1^12^ | 78.03(2) |
| O4^4^ | K1 | K1^1^ | 101.06(3) |  | K1^12^ | S2 | K1^8^ | 60.86(2) |
| O4^4^ | K1 | S1^5^ | 108.86(4) |  | O4 | S2 | Ca1 | 53.13(7) |
| O5^8^ | K1 | K1^1^ | 133.29(4) |  | O4^1^ | S2 | Ca1^13^ | 126.42(7) |
| O5^8^ | K1 | O1^6^ | 64.35(6) |  | O4^1^ | S2 | Ca1 | 53.13(7) |
| O5^8^ | K1 | O1W | 104.27(7) |  | O4 | S2 | Ca1^13^ | 126.42(7) |
| O5^8^ | K1 | O2^5^ | 78.00(7) |  | O4^1^ | S2 | K1^12^ | 117.03(8) |
| O5^8^ | K1 | O2^7^ | 143.84(7) |  | O4^1^ | S2 | K1^8^ | 69.16(8) |
| O5^8^ | K1 | O3^7^ | 166.37(7) |  | O4 | S2 | K1^12^ | 69.16(8) |
| O5^8^ | K1 | O3^8^ | 83.55(7) |  | O4 | S2 | K1^8^ | 117.03(8) |
| O5^8^ | K1 | O4^4^ | 45.09(6) |  | O4^1^ | S2 | O4 | 106.24(14) |
| O5^8^ | K1 | O6^2^ | 82.47(5) |  | O4^1^ | S2 | O5 | 109.42(10) |
| O5^8^ | K1 | S1^7^ | 162.30(5) |  | O4 | S2 | O5 | 109.42(10) |
| O5^8^ | K1 | S1^5^ | 64.85(5) |  | O5 | S2 | Ca1 | 124.82(11) |
| O6^2^ | K1 | K1^1^ | 50.94(4) |  | O5 | S2 | Ca1^13^ | 47.90(10) |
| O6^2^ | K1 | O1^6^ | 77.25(6) |  | O5 | S2 | K1^8^ | 40.53(7) |
| O6^2^ | K1 | O1W | 61.34(7) |  | O5 | S2 | K1^12^ | 40.53(7) |
| O6^2^ | K1 | O2^7^ | 89.82(6) |  | O6 | S2 | Ca1 | 127.54(11) |
| O6^2^ | K1 | O2^5^ | 126.15(7) |  | O6 | S2 | Ca1^13^ | 59.74(11) |
| O6^2^ | K1 | O3^8^ | 142.66(8) |  | O6 | S2 | K1^8^ | 128.20(9) |
| O6^2^ | K1 | O3^7^ | 104.55(5) |  | O6 | S2 | K1^12^ | 128.20(9) |
| O6^2^ | K1 | O4^4^ | 63.19(6) |  | O6 | S2 | O4 | 112.04(10) |
| O6^2^ | K1 | S1^7^ | 107.00(4) |  | O6 | S2 | O4^1^ | 112.04(10) |
| O6^2^ | K1 | S1^5^ | 102.08(5) |  | O6 | S2 | O5 | 107.64(15) |
| S1^7^ | K1 | K1^1^ | 58.783(12) |  |  |  |  |  |

^1^+X,1/2-Y,+Z; ^2^1+X,+Y,+Z; ^3^1-X,1-Y,1-Z; ^4^1-X,-1/2+Y,1-Z; ^5^2-X,-Y,1-Z; ^6^2-X,-1/2+Y,1-Z; ^7^+X,+Y,-1+Z; ^8^1-X,-Y,1-Z; ^9^2-X,1/2+Y,1-Z; ^10^+X,+Y,1+Z; ^11^+X,1/2-Y,1+Z; ^12^1-X,1/2+Y,1-Z; ^13^-1+X,+Y,+Z; ^14^-1+X,1/2-Y,+Z

**Table S10.** Values of torsion angles for syngenite [ º].

| **A** | **B** | **C** | **D** | **Angle/˚** |  | **A** | **B** | **C** | **D** | **Angle/˚** |
| --- | --- | --- | --- | --- | --- | --- | --- | --- | --- | --- |
| Ca1 | O1 | S1 | K1^1^ | 179.66(5) |  | K1^6^ | O2 | S1 | O1 | 159.20(9) |
| Ca1 | O1 | S1 | K1^2^ | -72.32(8) |  | K1^1^ | O2 | S1 | O3 | -37.43(4) |
| Ca1 | O1 | S1 | K1^3^ | -109.25(7) |  | K1^5^ | O2 | S1 | O3 | -140.17(5) |
| Ca1 | O1 | S1 | K1^4^ | 62.29(7) |  | K1^6^ | O2 | S1 | O3 | 37.43(4) |
| Ca1 | O1 | S1 | K1^5^ | 103.12(8) |  | K1^4^ | O2 | S1 | O3 | 140.17(5) |
| Ca1 | O1 | S1 | K1^6^ | 175.32(17) |  | K1^1^ | O3 | S1 | Ca1 | -144.16(3) |
| Ca1 | O1 | S1 | O1^7^ | 2.25(15) |  | K1^6^ | O3 | S1 | Ca1 | 144.16(4) |
| Ca1 | O1 | S1 | O2 | 121.13(11) |  | K1^2^ | O3 | S1 | Ca1 | 46.53(9) |
| Ca1 | O1 | S1 | O3 | -117.88(12) |  | K1^3^ | O3 | S1 | Ca1 | -46.53(9) |
| Ca1^8^ | O4 | S2 | Ca1 | 156.5(2) |  | K1^3^ | O3 | S1 | K1^5^ | 75.1(2) |
| Ca1^8^ | O4 | S2 | Ca1^9^ | -32.6(2) |  | K1^2^ | O3 | S1 | K1^5^ | 168.13(6) |
| Ca1 | O4 | S2 | Ca1^9^ | 170.94(5) |  | K1^3^ | O3 | S1 | K1^4^ | -168.13(6) |
| Ca1 | O4 | S2 | K1^2^ | 75.94(8) |  | K1^6^ | O3 | S1 | K1^4^ | 22.56(16) |
| Ca1^8^ | O4 | S2 | K1^2^ | -127.57(17) |  | K1^1^ | O3 | S1 | K1^4^ | 94.25(10) |
| Ca1 | O4 | S2 | K1^3^ | 114.76(7) |  | K1^1^ | O3 | S1 | K1^5^ | -22.56(16) |
| Ca1^8^ | O4 | S2 | K1^3^ | -88.76(19) |  | K1^6^ | O3 | S1 | K1^5^ | -94.25(10) |
| Ca1^8^ | O4 | S2 | O4^7^ | 157.94(11) |  | K1^3^ | O3 | S1 | K1^2^ | -93.06(18) |
| Ca1 | O4 | S2 | O4^7^ | 1.45(16) |  | K1^3^ | O3 | S1 | K1^1^ | 97.63(6) |
| Ca1 | O4 | S2 | O5 | 119.49(11) |  | K1^1^ | O3 | S1 | K1^6^ | 71.69(7) |
| Ca1^8^ | O4 | S2 | O5 | -84.0(2) |  | K1^2^ | O3 | S1 | K1^1^ | -169.32(12) |
| Ca1^8^ | O4 | S2 | O6 | 35.3(2) |  | K1^6^ | O3 | S1 | K1^1^ | -71.69(7) |
| Ca1 | O4 | S2 | O6 | -121.20(11) |  | K1^6^ | O3 | S1 | K1^3^ | -169.32(12) |
| Ca1^9^ | O5 | S2 | Ca1 | 180.000(0) |  | K1^2^ | O3 | S1 | K1^3^ | 93.06(18) |
| Ca1^9^ | O5 | S2 | K1^3^ | 128.80(9) |  | K1^2^ | O3 | S1 | K1^6^ | -97.63(6) |
| Ca1^9^ | O5 | S2 | K1^2^ | -128.80(9) |  | K1^1^ | O3 | S1 | K1^2^ | 169.32(12) |
| Ca1^9^ | O5 | S2 | O4 | 121.99(9) |  | K1^2^ | O3 | S1 | K1^4^ | -75.1(2) |
| Ca1^9^ | O5 | S2 | O4^7^ | -121.99(9) |  | K1^3^ | O3 | S1 | K1^6^ | 169.32(12) |
| Ca1^9^ | O5 | S2 | O6 | 0.000(1) |  | K1^6^ | O3 | S1 | K1^2^ | 97.63(6) |
| Ca1^9^ | O6 | S2 | Ca1 | 180.000(0) |  | K1^1^ | O3 | S1 | K1^3^ | -97.63(6) |
| Ca1^9^ | O6 | S2 | K1^3^ | -40.13(6) |  | K1^2^ | O3 | S1 | O1 | 105.19(10) |
| Ca1^9^ | O6 | S2 | K1^2^ | 40.13(6) |  | K1^1^ | O3 | S1 | O1 | -85.49(11) |
| Ca1^9^ | O6 | S2 | O4^7^ | 120.35(9) |  | K1^3^ | O3 | S1 | O1 | 12.13(15) |
| Ca1^9^ | O6 | S2 | O4 | -120.35(9) |  | K1^1^ | O3 | S1 | O1^7^ | 157.18(9) |
| Ca1^9^ | O6 | S2 | O5 | 0.000(1) |  | K1^6^ | O3 | S1 | O1^7^ | 85.49(11) |
| K1^5^ | O1 | S1 | Ca1 | -103.12(8) |  | K1^6^ | O3 | S1 | O1 | -157.18(9) |
| K1^5^ | O1 | S1 | K1^1^ | 76.53(5) |  | K1^2^ | O3 | S1 | O1^7^ | -12.13(15) |
| K1^5^ | O1 | S1 | K1^6^ | 72.2(2) |  | K1^3^ | O3 | S1 | O1^7^ | -105.19(10) |
| K1^5^ | O1 | S1 | K1^4^ | -40.83(7) |  | K1^3^ | O3 | S1 | O2 | 133.47(9) |
| K1^5^ | O1 | S1 | K1^2^ | -175.45(3) |  | K1^1^ | O3 | S1 | O2 | 35.84(3) |
| K1^5^ | O1 | S1 | K1^3^ | 147.63(7) |  | K1^2^ | O3 | S1 | O2 | -133.47(9) |
| K1^5^ | O1 | S1 | O1^7^ | -100.87(11) |  | K1^6^ | O3 | S1 | O2 | -35.84(4) |
| K1^5^ | O1 | S1 | O2 | 18.01(13) |  | K1^3^ | O4 | S2 | Ca1^9^ | 56.18(8) |
| K1^5^ | O1 | S1 | O3 | 139.00(11) |  | K1^3^ | O4 | S2 | Ca1 | -114.76(7) |
| K1^4^ | O2 | S1 | Ca1 | -39.83(5) |  | K1^3^ | O4 | S2 | K1^2^ | -38.82(6) |
| K1^6^ | O2 | S1 | Ca1 | -142.57(4) |  | K1^3^ | O4 | S2 | O4^7^ | -113.30(10) |
| K1^1^ | O2 | S1 | Ca1 | 142.57(4) |  | K1^3^ | O4 | S2 | O5 | 4.73(11) |
| K1^5^ | O2 | S1 | Ca1 | 39.83(5) |  | K1^3^ | O4 | S2 | O6 | 124.04(11) |
| K1^1^ | O2 | S1 | K1^5^ | 102.742(16) |  | K1^2^ | O5 | S2 | Ca1^9^ | 128.80(9) |
| K1^6^ | O2 | S1 | K1^3^ | 78.95(4) |  | K1^3^ | O5 | S2 | Ca1^9^ | -128.80(9) |
| K1^1^ | O2 | S1 | K1^6^ | -74.85(8) |  | K1^3^ | O5 | S2 | Ca1 | 51.20(9) |
| K1^5^ | O2 | S1 | K1^6^ | -177.60(8) |  | K1^2^ | O5 | S2 | Ca1 | -51.20(9) |
| K1^1^ | O2 | S1 | K1^4^ | -177.60(9) |  | K1^2^ | O5 | S2 | K1^3^ | -102.40(19) |
| K1^5^ | O2 | S1 | K1^3^ | -98.64(12) |  | K1^3^ | O5 | S2 | K1^2^ | 102.40(18) |
| K1^4^ | O2 | S1 | K1^5^ | -79.66(9) |  | K1^3^ | O5 | S2 | O4^7^ | 109.22(10) |
| K1^6^ | O2 | S1 | K1^5^ | 177.60(9) |  | K1^3^ | O5 | S2 | O4 | -6.81(15) |
| K1^5^ | O2 | S1 | K1^4^ | 79.66(9) |  | K1^2^ | O5 | S2 | O4^7^ | 6.81(15) |
| K1^5^ | O2 | S1 | K1^2^ | 178.30(4) |  | K1^2^ | O5 | S2 | O4 | -109.22(10) |
| K1^4^ | O2 | S1 | K1^6^ | 102.742(16) |  | K1^3^ | O5 | S2 | O6 | -128.80(9) |
| K1^5^ | O2 | S1 | K1^1^ | -102.742(16) |  | K1^2^ | O5 | S2 | O6 | 128.80(9) |
| K1^4^ | O2 | S1 | K1^1^ | 177.60(9) |  | K1^9^ | O6 | S2 | Ca1^9^ | -111.07(18) |
| K1^4^ | O2 | S1 | K1^2^ | 98.64(12) |  | K1^10^ | O6 | S2 | Ca1 | -68.93(18) |
| K1^6^ | O2 | S1 | K1^1^ | 74.85(8) |  | K1^9^ | O6 | S2 | Ca1 | 68.93(18) |
| K1^6^ | O2 | S1 | K1^4^ | -102.742(16) |  | K1^10^ | O6 | S2 | Ca1^9^ | 111.07(18) |
| K1^1^ | O2 | S1 | K1^3^ | 4.10(11) |  | K1^10^ | O6 | S2 | K1^3^ | 70.9(2) |
| K1^1^ | O2 | S1 | K1^2^ | -78.95(4) |  | K1^9^ | O6 | S2 | K1^2^ | -70.9(2) |
| K1^4^ | O2 | S1 | K1^3^ | -178.30(4) |  | K1^9^ | O6 | S2 | K1^3^ | -151.20(13) |
| K1^6^ | O2 | S1 | K1^2^ | -4.10(11) |  | K1^10^ | O6 | S2 | K1^2^ | 151.20(13) |
| K1^1^ | O2 | S1 | O1 | 84.35(11) |  | K1^9^ | O6 | S2 | O4^7^ | 9.3(2) |
| K1^5^ | O2 | S1 | O1 | -18.40(12) |  | K1^9^ | O6 | S2 | O4 | 128.58(17) |
| K1^4^ | O2 | S1 | O1 | -98.06(9) |  | K1^10^ | O6 | S2 | O4 | -9.3(2) |
| K1^6^ | O2 | S1 | O1^7^ | -84.35(11) |  | K1^10^ | O6 | S2 | O4^7^ | -128.58(17) |
| K1^5^ | O2 | S1 | O1^7^ | 98.06(9) |  | K1^9^ | O6 | S2 | O5 | -111.07(18) |
| K1^4^ | O2 | S1 | O1^7^ | 18.39(12) |  | K1^10^ | O6 | S2 | O5 | 111.07(18) |
| K1^1^ | O2 | S1 | O1^7^ | -159.20(9) |  |  |  |  |  |  |

^1^+X,1/2-Y,1+Z; ^2^1-X,-Y,1-Z; ^3^1-X,1/2+Y,1-Z; ^4^2-X,-Y,1-Z; ^5^2-X,1/2+Y,1-Z; ^6^+X,+Y,1+Z; ^7^+X,1/2-Y,+Z; ^8^1-X,1-Y,1-Z; ^9^-1+X,+Y,+Z; ^10^-1+X,1/2-Y,+Z

**Table S11.** Bond lengths for picromerite [Å].

| **Atom** | **Atom** | **Length/Å** |  | **Atom** | **Atom** | **Length/Å** |
| --- | --- | --- | --- | --- | --- | --- |
| Mg1 | O1W^1^ | 2.1007(12) |  | K1 | O3^7^ | 2.8485(14) |
| Mg1 | O1W | 2.1007(12) |  | K1 | O4^8^ | 2.8016(13) |
| Mg1 | O2W^1^ | 2.0172(13) |  | K1 | S1^6^ | 3.4343(6) |
| Mg1 | O2W | 2.0172(13) |  | K1 | S1 | 3.5743(5) |
| Mg1 | O3W^1^ | 2.0912(12) |  | O1 | S1 | 1.4800(13) |
| Mg1 | O3W | 2.0912(12) |  | O2 | K1^6^ | 2.8981(14) |
| O1W | K1^2^ | 2.9718(13) |  | O2 | S1 | 1.4697(13) |
| O3W | K1^3^ | 3.0850(13) |  | O3 | K1^9^ | 2.8485(14) |
| K1 | O1W^4^ | 2.9717(13) |  | O3 | K1^6^ | 2.9028(13) |
| K1 | O3W^5^ | 3.0850(13) |  | O3 | S1 | 1.4827(13) |
| K1 | O1 | 2.7045(13) |  | O4 | K1^8^ | 2.8016(13) |
| K1 | O2 | 3.3091(14) |  | O4 | S1 | 1.4843(12) |
| K1 | O2^6^ | 2.8981(14) |  | S1 | K1^6^ | 3.4343(6) |
| K1 | O3^6^ | 2.9028(13) |  |  |  |  |

^1^1-X,2-Y,1-Z; ^2^1-X,1/2+Y,1/2-Z; ^3^+X,1+Y,+Z; ^4^1-X,-1/2+Y,1/2-Z; ^5^+X,-1+Y,+Z; ^6^-X,1-Y,1-Z; ^7^-X,-1/2+Y,1/2-Z; ^8^1-X,1-Y,1-Z; ^9^-X,1/2+Y,1/2-Z

**Table S12.** Values of valence angles for picromerite [ º].

| **Atom** | **Atom** | **Atom** | **Angle/˚** |  | **Atom** | **Atom** | **Atom** | **Angle/˚** |
| --- | --- | --- | --- | --- | --- | --- | --- | --- |
| O1W | Mg1 | O1W^1^ | 180.00(9) |  | O3^7^ | K1 | O3W^5^ | 64.49(3) |
| O2W | Mg1 | O1W | 89.39(5) |  | O3^6^ | K1 | O3W^5^ | 92.47(4) |
| O2W | Mg1 | O1W^1^ | 90.61(5) |  | O3^7^ | K1 | O2^6^ | 95.43(4) |
| O2W^1^ | Mg1 | O1W^1^ | 89.39(5) |  | O3^7^ | K1 | O2 | 147.77(4) |
| O2W^1^ | Mg1 | O1W | 90.61(5) |  | O3^6^ | K1 | O2 | 86.12(3) |
| O2W | Mg1 | O2W^1^ | 180.0 |  | O3^7^ | K1 | O3^6^ | 104.27(3) |
| O2W | Mg1 | O3W | 90.44(5) |  | O3^6^ | K1 | S1^6^ | 25.32(3) |
| O2W^1^ | Mg1 | O3W^1^ | 90.44(5) |  | O3^7^ | K1 | S1^6^ | 106.26(3) |
| O2W | Mg1 | O3W^1^ | 89.56(5) |  | O3^7^ | K1 | S1 | 126.22(3) |
| O2W^1^ | Mg1 | O3W | 89.56(5) |  | O3^6^ | K1 | S1 | 106.03(3) |
| O3W^1^ | Mg1 | O1W^1^ | 90.35(5) |  | O4^8^ | K1 | O1W^4^ | 85.19(4) |
| O3W | Mg1 | O1W^1^ | 89.65(5) |  | O4^8^ | K1 | O3W^5^ | 76.88(4) |
| O3W^1^ | Mg1 | O1W | 89.65(5) |  | O4^8^ | K1 | O2 | 69.83(4) |
| O3W | Mg1 | O1W | 90.35(5) |  | O4^8^ | K1 | O2^6^ | 118.14(4) |
| O3W | Mg1 | O3W^1^ | 180.0 |  | O4^8^ | K1 | O3^6^ | 85.16(4) |
| Mg1 | O1W | K1^2^ | 137.71(5) |  | O4^8^ | K1 | O3^7^ | 140.37(4) |
| Mg1 | O3W | K1^3^ | 128.09(5) |  | O4^8^ | K1 | S1^6^ | 98.30(3) |
| O1W^4^ | K1 | O3W^5^ | 78.52(3) |  | O4^8^ | K1 | S1 | 85.67(3) |
| O1W^4^ | K1 | O2 | 97.08(3) |  | S1^6^ | K1 | S1 | 85.511(13) |
| O1W^4^ | K1 | S1^6^ | 164.99(3) |  | S1 | O1 | K1 | 114.12(7) |
| O1W^4^ | K1 | S1 | 80.18(3) |  | K1^6^ | O2 | K1 | 111.86(4) |
| O3W^5^ | K1 | O2 | 146.68(4) |  | S1 | O2 | K1^6^ | 98.34(7) |
| O3W^5^ | K1 | S1^6^ | 116.48(3) |  | S1 | O2 | K1 | 88.03(6) |
| O3W^5^ | K1 | S1 | 153.41(3) |  | K1^9^ | O3 | K1^6^ | 122.48(5) |
| O1 | K1 | O1W^4^ | 72.32(4) |  | S1 | O3 | K1^6^ | 97.81(6) |
| O1 | K1 | O3W^5^ | 150.36(4) |  | S1 | O3 | K1^9^ | 126.12(7) |
| O1 | K1 | O2 | 45.69(4) |  | S1 | O4 | K1^8^ | 122.95(7) |
| O1 | K1 | O2^6^ | 73.23(4) |  | K1^6^ | S1 | K1 | 94.489(13) |
| O1 | K1 | O3^6^ | 117.14(4) |  | O1 | S1 | K1^6^ | 105.96(5) |
| O1 | K1 | O3^7^ | 104.02(4) |  | O1 | S1 | K1 | 43.68(5) |
| O1 | K1 | O4^8^ | 105.39(4) |  | O1 | S1 | O3 | 108.11(7) |
| O1 | K1 | S1^6^ | 92.70(3) |  | O1 | S1 | O4 | 110.06(7) |
| O1 | K1 | S1 | 22.21(3) |  | O2 | S1 | K1 | 67.71(6) |
| O2^6^ | K1 | O1W^4^ | 142.51(4) |  | O2 | S1 | K1^6^ | 56.61(6) |
| O2^6^ | K1 | O3W^5^ | 132.62(4) |  | O2 | S1 | O1 | 108.93(8) |
| O2^6^ | K1 | O2 | 68.14(4) |  | O2 | S1 | O3 | 109.77(8) |
| O2^6^ | K1 | O3^6^ | 49.20(4) |  | O2 | S1 | O4 | 110.22(8) |
| O2^6^ | K1 | S1 | 73.43(3) |  | O3 | S1 | K1 | 136.21(5) |
| O2 | K1 | S1^6^ | 70.79(2) |  | O3 | S1 | K1^6^ | 56.87(5) |
| O2^6^ | K1 | S1^6^ | 25.05(3) |  | O3 | S1 | O4 | 109.72(7) |
| O2 | K1 | S1 | 24.26(2) |  | O4 | S1 | K1^6^ | 143.97(5) |
| O3^6^ | K1 | O1W^4^ | 168.07(4) |  | O4 | S1 | K1 | 111.80(5) |
| O3^7^ | K1 | O1W^4^ | 79.06(4) |  |  |  |  |  |

^1^1-X,2-Y,1-Z; ^2^1-X,1/2+Y,1/2-Z; ^3^+X,1+Y,+Z; ^4^1-X,-1/2+Y,1/2-Z; ^5^+X,-1+Y,+Z; ^6^-X,1-Y,1-Z; ^7^-X,-1/2+Y,1/2-Z; ^8^1-X,1-Y,1-Z; ^9^-X,1/2+Y,1/2-Z

**Table S13.** Values of torsion angles for picromerite [ º].

| **A** | **B** | **C** | **D** | **Angle/˚** |  | **A** | **B** | **C** | **D** | **Angle/˚** |
| --- | --- | --- | --- | --- | --- | --- | --- | --- | --- | --- |
| K1 | O1 | S1 | K1^1^ | -79.53(6) |  | K1^2^ | O3 | S1 | K1^1^ | -140.45(9) |
| K1 | O1 | S1 | O2 | -20.00(9) |  | K1^1^ | O3 | S1 | K1 | 56.91(8) |
| K1 | O1 | S1 | O3 | -139.23(7) |  | K1^2^ | O3 | S1 | O1 | -42.86(10) |
| K1 | O1 | S1 | O4 | 100.95(8) |  | K1^1^ | O3 | S1 | O1 | 97.59(7) |
| K1^1^ | O2 | S1 | K1 | -111.81(5) |  | K1^2^ | O3 | S1 | O2 | -161.56(8) |
| K1 | O2 | S1 | K1^1^ | 111.81(5) |  | K1^1^ | O3 | S1 | O2 | -21.11(8) |
| K1 | O2 | S1 | O1 | 14.79(7) |  | K1^1^ | O3 | S1 | O4 | -142.37(6) |
| K1^1^ | O2 | S1 | O1 | -97.02(7) |  | K1^2^ | O3 | S1 | O4 | 77.18(9) |
| K1 | O2 | S1 | O3 | 132.98(6) |  | K1^3^ | O4 | S1 | K1 | -80.66(7) |
| K1^1^ | O2 | S1 | O3 | 21.17(8) |  | K1^3^ | O4 | S1 | K1^1^ | 53.22(12) |
| K1^1^ | O2 | S1 | O4 | 142.13(6) |  | K1^3^ | O4 | S1 | O1 | -127.57(8) |
| K1 | O2 | S1 | O4 | -106.06(6) |  | K1^3^ | O4 | S1 | O2 | -7.40(10) |
| K1^2^ | O3 | S1 | K1 | -83.54(9) |  | K1^3^ | O4 | S1 | O3 | 113.59(8) |

^1^-X,1-Y,1-Z; ^2^-X,1/2+Y,1/2-Z; ^3^1-X,1-Y,1-Z

**Table S14.** Bond lengths for subhydrate CaSO_4_·0.69H_2_O [Å].

| **Atom** | **Atom** | **Length/Å** |  | **Atom** | **Atom** | **Length/Å** |
| --- | --- | --- | --- | --- | --- | --- |
| Ca1 | Ca1^1^ | 4.0958(4) |  | Ca1 | S1^7^ | 3.17317(18) |
| Ca1 | O1^2^ | 2.503(3) |  | O1 | Ca1^1^ | 2.503(3) |
| Ca1 | O1^3^ | 2.423(4) |  | O1 | S1 | 1.456(3) |
| Ca1 | O1 | 2.423(4) |  | O2 | Ca1^8^ | 2.442(4) |
| Ca1 | O1^4^ | 2.503(3) |  | O2 | Ca1^1^ | 2.645(4) |
| Ca1 | O1W | 2.503(9) |  | O2 | S1 | 1.466(3) |
| Ca1 | O2^5^ | 2.442(4) |  | S1 | Ca1^1^ | 3.17317(17) |
| Ca1 | O2^2^ | 2.645(4) |  | S1 | Ca1^9^ | 3.17321(17) |
| Ca1 | O2^6^ | 2.442(4) |  | S1 | O1^10^ | 1.456(3) |
| Ca1 | O2^4^ | 2.645(4) |  | S1 | O2^10^ | 1.466(3) |
| Ca1 | S1^2^ | 3.17321(17) |  |  |  |  |

^1^1+Y-X,1-X,1/3+Z; ^2^1-Y,+X-Y,-1/3+Z; ^3^-Y+X,-Y,4/3-Z; ^4^1-X,-X+Y,5/3-Z; ^5^1-Y+X,1-Y,4/3-Z; ^6^+X,-1+Y,+Z; ^7^1-Y,+X-Y,2/3+Z; ^8^+X,1+Y,+Z; ^9^1+Y-X,1-X,-2/3+Z; ^10^+Y,+X,1-Z

**Table S15.** Values of valence angles for CaSO_4_·0.69H_2_O [ º].

| **Atom** | **Atom** | **Atom** | **Angle/˚** |  | **Atom** | **Atom** | **Atom** | **Angle/˚** |
| --- | --- | --- | --- | --- | --- | --- | --- | --- |
| O1 | Ca1 | Ca1^1^ | 34.38(7) |  | O2^5^ | Ca1 | O1^4^ | 69.37(12) |
| O1^2^ | Ca1 | Ca1^1^ | 108.34(8) |  | O2^5^ | Ca1 | O1^2^ | 82.67(13) |
| O1^3^ | Ca1 | Ca1^1^ | 124.78(12) |  | O2^6^ | Ca1 | O1^4^ | 82.67(13) |
| O1^4^ | Ca1 | Ca1^1^ | 83.06(7) |  | O2^6^ | Ca1 | O1W | 140.23(8) |
| O1^3^ | Ca1 | O1^2^ | 117.30(9) |  | O2^5^ | Ca1 | O1W | 140.23(8) |
| O1 | Ca1 | O1^2^ | 78.32(13) |  | O2^5^ | Ca1 | O2^2^ | 87.89(13) |
| O1^4^ | Ca1 | O1^2^ | 143.6(2) |  | O2^6^ | Ca1 | O2^5^ | 79.55(16) |
| O1 | Ca1 | O1^4^ | 117.30(9) |  | O2^6^ | Ca1 | O2^4^ | 87.89(13) |
| O1^3^ | Ca1 | O1^4^ | 78.32(13) |  | O2^5^ | Ca1 | O2^4^ | 122.91(10) |
| O1^3^ | Ca1 | O1 | 131.6(2) |  | O2^6^ | Ca1 | O2^2^ | 122.91(10) |
| O1 | Ca1 | O1W | 65.78(11) |  | O2^4^ | Ca1 | O2^2^ | 141.52(16) |
| O1^3^ | Ca1 | O1W | 65.78(11) |  | O2^4^ | Ca1 | S1^7^ | 27.30(7) |
| O1 | Ca1 | O2^2^ | 96.63(11) |  | O2^6^ | Ca1 | S1^7^ | 85.20(9) |
| O1 | Ca1 | O2^5^ | 152.43(14) |  | O2^2^ | Ca1 | S1^7^ | 151.78(9) |
| O1^4^ | Ca1 | O2^2^ | 142.79(10) |  | O2^2^ | Ca1 | S1^2^ | 27.29(7) |
| O1 | Ca1 | O2^6^ | 75.18(13) |  | O2^4^ | Ca1 | S1^2^ | 151.78(9) |
| O1^3^ | Ca1 | O2^4^ | 96.63(11) |  | O2^5^ | Ca1 | S1^7^ | 95.81(9) |
| O1^3^ | Ca1 | O2^6^ | 152.43(14) |  | O2^6^ | Ca1 | S1^2^ | 95.81(9) |
| O1 | Ca1 | O2^4^ | 67.30(11) |  | O2^5^ | Ca1 | S1^2^ | 85.20(9) |
| O1^3^ | Ca1 | O2^5^ | 75.18(13) |  | S1^7^ | Ca1 | Ca1^1^ | 58.669(17) |
| O1^4^ | Ca1 | O2^4^ | 53.80(11) |  | S1^2^ | Ca1 | Ca1^1^ | 120.855(13) |
| O1^3^ | Ca1 | O2^2^ | 67.30(11) |  | S1^7^ | Ca1 | S1^2^ | 178.69(8) |
| O1^2^ | Ca1 | O2^2^ | 53.80(11) |  | Ca1 | O1 | Ca1^1^ | 112.50(12) |
| O1^2^ | Ca1 | O2^4^ | 142.79(10) |  | S1 | O1 | Ca1^1^ | 103.35(19) |
| O1^3^ | Ca1 | S1^7^ | 86.56(7) |  | S1 | O1 | Ca1 | 142.45(19) |
| O1^4^ | Ca1 | S1^7^ | 26.51(8) |  | Ca1^8^ | O2 | Ca1^1^ | 107.18(11) |
| O1^2^ | Ca1 | S1^7^ | 154.42(9) |  | S1 | O2 | Ca1^1^ | 96.84(19) |
| O1 | Ca1 | S1^2^ | 86.56(7) |  | S1 | O2 | Ca1^8^ | 136.8(2) |
| O1^3^ | Ca1 | S1^2^ | 92.90(7) |  | Ca1^1^ | S1 | Ca1^9^ | 178.69(8) |
| O1^2^ | Ca1 | S1^2^ | 26.51(8) |  | O1 | S1 | Ca1^1^ | 50.14(14) |
| O1 | Ca1 | S1^7^ | 92.90(7) |  | O1^10^ | S1 | Ca1^1^ | 128.91(15) |
| O1^4^ | Ca1 | S1^2^ | 154.42(9) |  | O1^10^ | S1 | Ca1^9^ | 50.14(14) |
| O1W | Ca1 | Ca1^1^ | 71.91(4) |  | O1 | S1 | Ca1^9^ | 128.91(15) |
| O1W | Ca1 | O1^4^ | 108.20(10) |  | O1 | S1 | O1^10^ | 111.6(3) |
| O1W | Ca1 | O1^2^ | 108.20(10) |  | O1 | S1 | O2^10^ | 111.43(18) |
| O1W | Ca1 | O2^2^ | 70.76(8) |  | O1^10^ | S1 | O2^10^ | 106.0(2) |
| O1W | Ca1 | O2^4^ | 70.76(8) |  | O1^10^ | S1 | O2 | 111.43(18) |
| O1W | Ca1 | S1^2^ | 89.34(4) |  | O1 | S1 | O2 | 106.0(2) |
| O1W | Ca1 | S1^7^ | 89.34(4) |  | O2 | S1 | Ca1^1^ | 55.86(15) |
| O2^2^ | Ca1 | Ca1^1^ | 128.12(8) |  | O2 | S1 | Ca1^9^ | 125.04(16) |
| O2^4^ | Ca1 | Ca1^1^ | 34.72(8) |  | O2^10^ | S1 | Ca1^9^ | 55.86(15) |
| O2^6^ | Ca1 | Ca1^1^ | 71.69(8) |  | O2^10^ | S1 | Ca1^1^ | 125.04(16) |
| O2^5^ | Ca1 | Ca1^1^ | 142.34(9) |  | O2 | S1 | O2^10^ | 110.5(3) |
| O2^6^ | Ca1 | O1^2^ | 69.37(12) |  |  |  |  |  |

^1^1+Y-X,1-X,1/3+Z; ^2^1-Y,+X-Y,-1/3+Z; ^3^-Y+X,-Y,4/3-Z; ^4^1-X,-X+Y,5/3-Z; ^5^+X,-1+Y,+Z; ^6^1-Y+X,1-Y,4/3-Z; ^7^1-Y,+X-Y,2/3+Z; ^8^+X,1+Y,+Z; ^9^1+Y-X,1-X,-2/3+Z; ^10^+Y,+X,1-Z

**Table S16.** Values of torsion angles for CaSO_4_·069H_2_O [ º].

| **A** | **B** | **C** | **D** | **Angle/˚** |  | **A** | **B** | **C** | **D** | **Angle/˚** |
| --- | --- | --- | --- | --- | --- | --- | --- | --- | --- | --- |
| Ca1 | O1 | S1 | Ca1^1^ | -162.5(5) |  | Ca1^4^ | O2 | S1 | Ca1^2^ | 54.6(3) |
| Ca1 | O1 | S1 | Ca1^2^ | 18.6(5) |  | Ca1^4^ | O2 | S1 | Ca1^1^ | -124.2(3) |
| Ca1^1^ | O1 | S1 | Ca1^2^ | -178.84(7) |  | Ca1^1^ | O2 | S1 | Ca1^2^ | 178.85(7) |
| Ca1^1^ | O1 | S1 | O1^3^ | -123.31(19) |  | Ca1^1^ | O2 | S1 | O1 | 1.7(2) |
| Ca1 | O1 | S1 | O1^3^ | 74.1(4) |  | Ca1^1^ | O2 | S1 | O1^3^ | 123.31(19) |
| Ca1^1^ | O1 | S1 | O2^3^ | 118.41(19) |  | Ca1^4^ | O2 | S1 | O1 | -122.6(3) |
| Ca1^1^ | O1 | S1 | O2 | -1.8(2) |  | Ca1^4^ | O2 | S1 | O1^3^ | -0.9(4) |
| Ca1 | O1 | S1 | O2^3^ | -44.1(5) |  | Ca1^1^ | O2 | S1 | O2^3^ | -119.16(17) |
| Ca1 | O1 | S1 | O2 | -164.4(4) |  | Ca1^4^ | O2 | S1 | O2^3^ | 116.6(3) |

^1^1+Y-X,1-X,1/3+Z; ^2^1+Y-X,1-X,-2/3+Z; ^3^+Y,+X,1-Z; ^4^+X,1+Y,+Z
